# The Cytokinome Profile in Patients with Hepatocellular Carcinoma and Type 2 Diabetes

**DOI:** 10.1371/journal.pone.0134594

**Published:** 2015-07-30

**Authors:** Francesca Capone, Eliana Guerriero, Giovanni Colonna, Patrizia Maio, Alessandra Mangia, Raffaele Marfella, Giuseppe Paolisso, Francesco Izzo, Nicoletta Potenza, Luigi Tomeo, Giuseppe Castello, Susan Costantini

**Affiliations:** 1 CROM, Istituto Nazionale Tumori “Fondazione G. Pascale”—IRCCS, Naples, Italy; 2 Center of Medical Informatics–SIM/AOU—Second University of Naples, Naples, Italy; 3 Unita`Operativa Malattie Infettive, Azienda Ospedaliera di Rilievo Nazionale ‘‘San Giuseppe Moscati”, Avellino, Italy; 4 Liver Unit, IRCCS Casa Sollievo della Sofferenza Hospital, San Giovanni Rotondo, Italy; 5 Department of Geriatrics and Metabolic Diseases, Second University of Naples, Naples, Italy; 6 Istituto Nazionale Tumori “Fondazione G. Pascale”—IRCCS, Naples, Italy; 7 Department of Environmental, Biological and Pharmaceutical Sciences and Technologies, Second University of Naples, Caserta, Italy; 8 Laboratorio Borriello SAS, Avellino, Italy; Kaohsiung Medical University Hospital, Kaohsiung Medical University, TAIWAN

## Abstract

Understanding the dynamics of the complex interaction network of cytokines, defined as ‘‘cytokinome’’, can be useful to follow progression and evolution of hepatocellular carcinoma (HCC) from its early stages as well as to define therapeutic strategies. Recently we have evaluated the cytokinome profile in patients with type 2 diabetes (T2D) and/or chronic hepatitis C (CHC) infection and/or cirrhosis suggesting specific markers for the different stages of the diseases. Since T2D has been identified as one of the contributory cause of HCC, in this paper we examined the serum levels of cytokines, growth factors, chemokines, as well as of other cancer and diabetes biomarkers in a discovery cohort of patients with T2D, chronic hepatitis C (CHC) and/or CHC-related HCC comparing them with a healthy control group to define a profile of proteins able to characterize these patients, and to recognize the association between diabetes and HCC. The results have evidenced that the serum levels of some proteins are significantly and differently up-regulated in all the patients but they increased still more when HCC develops on the background of T2D. Our results were verified also using a separate validation cohort. Furthermore, significant correlations between clinical and laboratory data characterizing the various stages of this complex disease, have been found. In overall, our results highlighted that a large and simple omics approach, such as that of the cytokinome analysis, supplemented by common biochemical and clinical data, can give a complete picture able to improve the prognosis of the various stages of the disease progression. We have also demonstrated by means of interactomic analysis that our experimental results correlate positively with the general metabolic picture that is emerging in the literature for this complex multifactorial disease.

## Introduction

Recently it has been reported that the liver cancer is the second death cause due to cancer. In particular, the hepatocellular carcinoma (HCC) is the more common form of liver cancer and are diagnosed more than 700,000 cases in each year [1]. Several risk factors have been identified to contribute to the international burden of HCC such as chronic infection with hepatitis B virus (HBV) and hepatitis C virus (HCV), alcoholic liver disease, non-alcoholic steato-hepatitis (NASH), diabetes mellitus (DM), obesity, intake of aflatoxins-contaminated food, tobacco smoking, excessive alcohol drinking and genetically inherited disorders (hemochromatosis, α-1 anti-trypsin deficiency, porphyrias) [2].

The type 2 diabetes (T2D) is a metabolic disorder characterized by hyperglycemia which may predispose the liver to relative insulin resistance due to inadequate secretion or receptor insensitivity to the endogenous insulin. In recent years, type 2 diabetes has been associated with increase risk for several malignancies including breast, colon, kidney, liver, endometrium and pancreatic cancers [3]. Recently some reported showed that the T2D presence tends to increase the HCC development and induces a poor prognosis for these patients, in both presence or absence of cirrhosis [4]. Because the liver plays a crucial role in glucose metabolism, it is not surprising that T2D is an epiphenomenon of many chronic liver diseases such as chronic hepatitis, fatty liver, liver failure and cirrhosis [5]. In addition, T2D as part of the insulin resistance syndrome, has been implicated as a risk factor for non-alcoholic fatty liver disease (NAFLD), including its most severe form non-alcoholic steato-hepatitis (NASH), and has been identified as a cause of both cirrhosis and HCC [6].

An important feature of the progression of chronic liver disease as well in the early stages of cancer is the minimal presence of clinical manifestations, making subtle the disease. In this context the cytokines are known to play an important role not only in the mechanisms of insulin resistance and glucose disposal defects but also in the pathological processes occurring in the liver during viral infection. In fact, understanding in patients affected from cancers or other diseases the dynamics of the complex interaction network of cytokines [7–9], defined ‘‘cytokinome” [10], should be very useful to follow the disease progression and evolution from its early stages as well as to define therapeutic strategies by using systems biology approaches [7–9].

Recently we evaluated the serum levels of many cytokines, chemokines, adipokines and growth factors in patients with type 2 diabetes, chronic hepatitis C (CHC) infection, CHC-related cirrhosis, CHC and type 2 diabetes and CHC-related cirrhosis and type 2 diabetes by BioPlex assay [9]. Our data evidenced that the serum levels of some proteins were significantly up-regulated in all the patients, but unfortunately they were often high also in individuals affected by only one syndrome, thus this fact makes not clearly attributable the analytes when both diseases are associated. Therefore, we suggested specific markers for the different stages of the diseases, useful for the clinical monitoring of patients in regard to the progression from CHC to LC and from CHD to LCD [9].

However, since the T2D is one of the most common co-morbid illnesses found in HCC patients, which is significantly associated with the worsening of the HCC development, we have focused more efforts on the understanding of the mechanisms underlying the HCC onset as well its progression, particularly in diabetic patients to try to improve their already poor prognosis. Therefore, aim of this study is to examine the serum levels of cytokines, growth factors, chemokines, as well as of other cancer and diabetes biomarkers in the patients with T2D, CHC, CHC-related HCC alone or in presence of T2D, comparing them with a healthy control group to define a profile of proteins able to characterize these patients, also identifying in the same time some diagnostic/prognostic markers useful for recognizing the association between diabetes and HCC.

## Methods

### Patients

In this study we enrolled in the discovery step 17 patients with T2D (11 women, 6 men), 20 patients with CHC (10 women and 10 men), 34 patients with HCC (11 women, 23 men), 10 patients with T2D-HCC (4 women, 6 men), and 20 healthy controls (11 women, 9 men). In **Table 1** we report clinical characteristics and biochemical laboratory data of all the patients. The ADA criteria were used to classify patients with the T2D [11]: i) fasting plasma glucose 126 mg/dL (7.0 mmol/L) where fasting is defined as no caloric intake for at least 8 h or ii) symptoms of hyperglycemia and a casual plasma glucose 200 mg/dL (11.1 mmol/L) where casual is defined as any time of day without regard to time since last meal whereas the classic symptoms of hyperglycemia include polyuria, polydipsia, and unexplained weight loss, or iii) 2-h plasma glucose 200 mg/dL (11.1 mmol/L) during an OGTT where the test has been performed as described by the World Health Organization, using a glucose load containing the equivalent of 75 g anhydrous glucose dissolved in water. The patients with T2D were overweight with BMI values in the range between 25–29 kg/m^2^. The stage of fibrosis was assessed for the CHC patients according to the Ishak index [12]. In particular, F2 corresponds to fibrosis of the majority of portal tracts, F3 to fibrosis of the majority of portal tracts with occasional port-portal septa, and F4 to fibrosis of the majority of portal tracts with port-portal and port-central septa. Moreover, all HCC patients had HCV-related cirrhosis, and were non treated with drugs. In particular, the severity of cirrhosis was defined by Child-Pugh score and liver biopsies were performed only on patients with Child-Pugh score A. The patients with HCC had higher serum transaminase alanine aminotransferase (ALT) and aspartate aminotransferase (AST) levels compared to the control patients, as evaluated in the healthy donors. Finally, the patients with HCC and T2D had hyperglycemia.

**Table 1:** Clinical and laboratory data of patients with type 2 diabetes (T2D), chronic HCV (CHC), HCC with HCV-related cirrhosis (HCC), and HCC with HCV-related cirrhosis and type 2 diabetes (T2D+HCC) belonging to discovery set. The corresponding patients belonging to validation set are indicated as T2D^V^, CHC^V^, HCC^V^ and T2D+HCC^V^. We report the number of patients to whom the parameters refer. The related control ranges of the clinical data evaluated for the healthy donors, are also shown.

	T2D	T2D^V^	CHC	CHC^V^	HCC	HCC^V^	T2D+HCC	T2D+HCC^V^	Control range
**Age**	61.8±5.2	57.8±6.1	62.5±9.5	60.0±9.0	71.0±6.1	65.1±9	68.3±8.3	67.8±7.8	60.92
**Gender**	11M-6F	10M-10F	10M-10F	11M-9F	23M-11F	12M-8F	6M-4F	7M-3F	
**Glycemia (mg/dL)**	145.9±12.3	154.1±8.2	86.0±10.1	98.0±4.1	84.2±7.1	90.0±11.2	172.3±21.4	164.5±16.4	70–105
**AST (IU/L)**	31±4	28±3	71.4±2.3	61.8±6.2	104±3	90.5±5.5	74±26.2	63.7±5.2	5–40
**ALT (IU/L)**	33±6	30±5	121.1±8.6	100.8±5.2	100±4	102±6.0	105±10.4	100±12	7–56
**TotBilirubin (mg/dL)**	1.01±0.06	1.1±0.01	0.94±0.08	1.0±0.04	1.51±0.05	1.38±0.04	1.3±0.6	1.4±0.8	0.20–1.30
**Albumin (g/dL)**	4.2±0.9	4.0±0.7	4.01±0.07	3.9±0.4	3±0.3	3.1±0.04	2.8±0.2	2.5±0.1	3.5–5
**PLT (mL)**	198464±10221	220000±112	187413±7315	191635±5256	124534±2341	126254±5211	148333±34239	150000±28684	150000–400000
**BMI (kg/m** ^**2**^ **)**	24.4±0.8	24.3±0.2	24.2±0.9	23.8±1.2	23.2±3.2	24.0±0.4	23.3±1.5	23.9±1.3	18.9–24.9
**HCV–PCR RNA**	negative	negative	positive	positive	positive	positive	positive	positive	negative
**HCV genot**	negative	negative	1:11; 2:9	1:13; 2:7	1: 20; 2: 14	1:14; 2:6	1:4; 2: 6	1:8; 2:2	
**AFP (ng/mL)**	<10	<10	<10	<10	150±20	120±40	173±31	170±25	<10
**Child Pügh**					A:15; B:13;C:6	A:5; B:4;C:1	A:2; B:2;C:6	A:3; B: 3;C:4	
**Tumor invasion**					T1:10; T2:12; T3:12	T1: 4; T2:4; T3: 2	T1:3; T2:3; T3:4	T1:4; T2:4; T3: 2	

Moreover, we verified the results using a separate validation cohort of 90 age/gender matched subjects, including 20 patients with T2D, 20 patients with CHC, 20 with HCC and 10 with T2D-HCC, and 20 healthy control subjects. These subjects had clinical characteristics similar to those used in the discovery step, and no significant differences can be evidenced between two sets (**Table 1**).

For this study we obtained ethics approval from the ethics committee of our institution (Second University of Naples) and obtained written informed consent from all involved participants.

### Bio-Plex Assay

Blood samples were collected from a peripheral vein and kept on ice. Serum was collected by centrifugation (3,000 rpm for 10 min at 4°C), aliquoted, and stored at −80°C until analyzed. A multiplex biometric ELISA-based immunoassay, containing dyed microspheres conjugated with a monoclonal antibody specific for a target protein was used according to the manufacturer’s instructions (Bio-plex, Bio-Rad Lab., Inc., Hercules, CA, USA). Soluble molecules were measured using four commercially available kits: i) 21-plex immunoassay panel: IL-1α, IL-2R, IL-3, IL-12p40, IL-16, IL-18, CCL27, CXCL1, CXCL9, CXCL12, HGF, IFN-α2, LIF, MCP-3, M-CSF, MIF, β-NGF, SCF, SCGF-β, TNF-β, TRAIL; ii) 16-Plex panel: sEGFR, FGF-basic, Follistatin, G-CSF, HGF, sHER-2/neu, sIL-6Rα, Leptin, Osteopontin, PECAM-1, PDGF-AB/BB, Prolactin (PRL), SCF, sTIE-2, sVEGFR-1 (FLT1) and sVEGFR-2 (KDR); iii) 10-Plex panel: C-peptide, ghrelin, GIP, glp-1, glucagon (GCG), insulin, leptin (LEP), PAI-1, resistin, visfatin and iv) 2-Plex panel: adiponectin (ADIPOQ) and adipsin.

Each experiment was performed in duplicate using the same procedure described in our recent papers [7–9]. Serum levels of all proteins were determined using a Bio-Plex array reader (Luminex, Austin, TX) that quantifies multiplex immunoassays in a 96-well plate with very small fluid volumes. The analytes concentration was calculated using a standard curve, with software provided by the manufacturer (Bio-Plex Manager Software).

### Data Analysis and Statistics

To evaluate the differences between cytokine, chemokine adipokines, cancer biomarkers and growth factor ratios in the patients and healthy controls belonging to discovery and validation steps, we used the nonparametric Mann-Whitney U test by obtaining U test and P values, the Unparied t test by P value, t value, the number of degrees of freedom (df), the difference between the means, 95% confidence interval, and R squared, and F test by F value, degrees of freedom for the numerator (DFn) and for the denominator (Dfd) and P value. In particular p<0.05 is indicated with *, p<0.01 with **, and p<0.001 with ***. Moreover, the correlations between the cytokine levels and clinical/biochemical data were determined using the Pearson correlation coefficient. Values of p<0.05 were considered to be statistically significant. The statistical programs Prism 4 (GraphPad Software, San Diego, CA, USA) was employed.

### Functional and Interactomic studies

The Database for Annotation, Visualization and Integrated Discovery (DAVID) was used to classify proteins according to their biological processes, as well as the metabolic pathways in which they are involved [13]. Moreover, the network analysis between the most significant proteins was performed by Ingenuity Pathway Analysis (IPA).

### Anti-TP53 assay

Anti-p53 antibodies were detected with an ELISA test kit (Pharmacell, Paris, France) by using microtiter plates coated with recombinant wild-type human p53 protein (to detect specific anti-p53 antibodies) or with a control protein (to detect nonspecific anti-p53 interactions). A peroxidase-conjugated goat antihuman IgG bound to anti-p53 antibodies. The specific p53/anti-p53-conjugated complexes were revealed by the addition of a peroxidase substrate (TMB), resulting in a colorimetric reaction. The absorbance was read at 450 nm, and the anti-p53 levels were expressed in units/mL and categorized as positive when >0.90 units/mL and negative otherwise [14].

## Results

### Comparison between Patients with T2D, CHC or HCC and Healthy Donors

**Fig 1:**
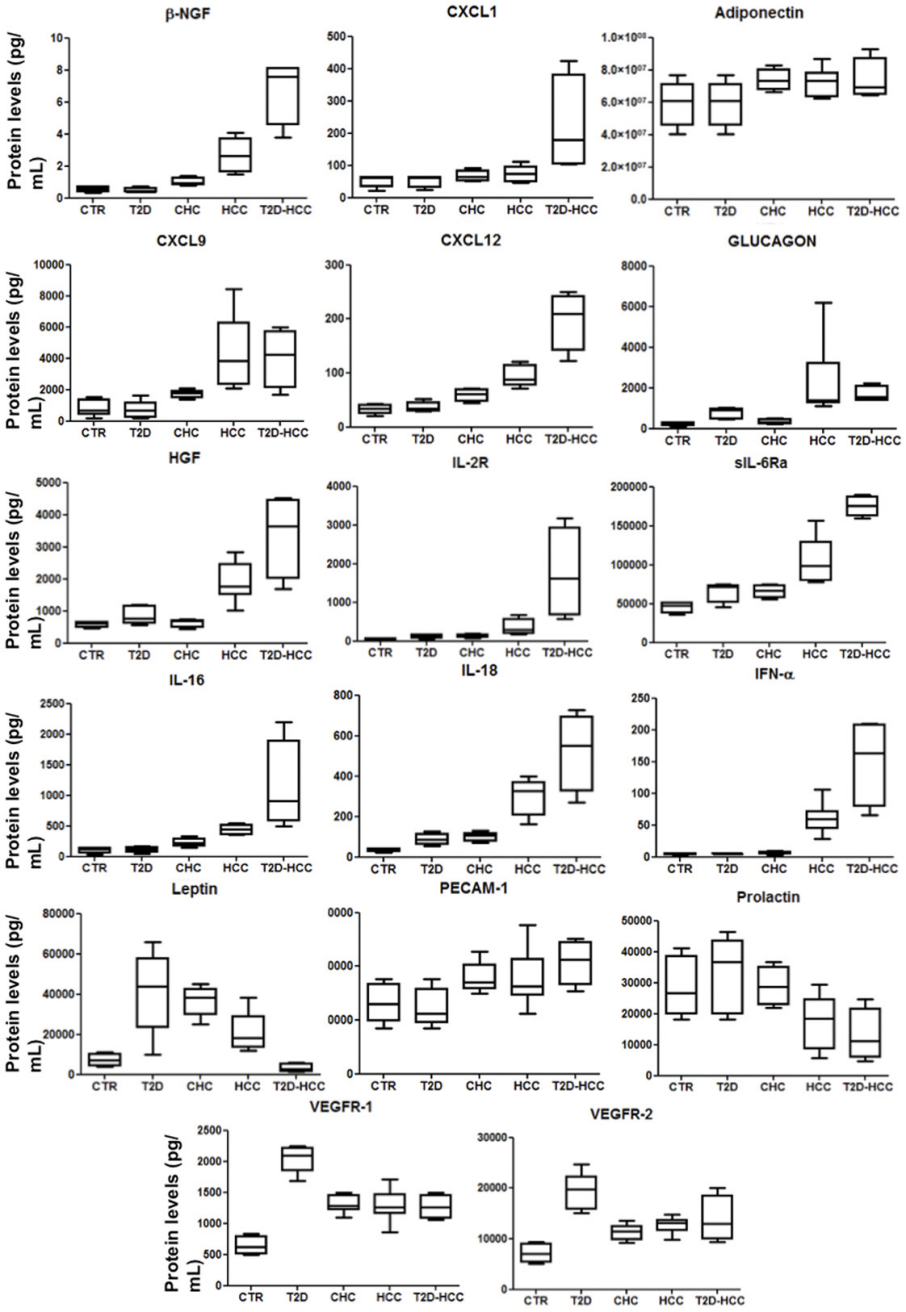
Significant cytokines in some patient groups belonging to discovery set. We report the significant molecule levels from controls, patients with type 2 diabetes (T2D), chronic hepatitis C (CHC), hepatocellular carcinoma (HCC) and hepatocellular carcinoma and type 2 diabetes (T2D-HCC) shown by means of box-and-whisker graphs. The boxes extend from the 25th to the 75th percentile, and the line in the middle is the median. The error bars extend down to the lowest value and up to the highest.

In Fig 1, and in Tables 2 and 3 we report the proteins that show different serum levels in T2D or CHC or HCC patients respect to controls with the related statistical evaluations; data not statistically significant are not reported. Greater amounts of HGF, IL-2R, s-IL-6Ra, IL-18, leptin, sVEGFR-1 and sVEGFR-2 were secreted by T2D, CHC and HCC patients in comparison with the healthy controls, whereas those of glucagon only by T2D and HCC patients, those of β-NGF, CXCL1, CXCL9, CXCL12, IL-16, and PECAM-1 by CHC and HCC patients, and those of IFN-α and Prolactin only in HCC patients. The most part of these data agrees with our recently published results. In fact, we have confirmed the increased amount of IL-2R, IL-18, HGF, glucagon, and leptin that were found in T2D patients [9] as well as of β-NGF, CXCL1, CXCL9, CXCL12, HGF, IL-2R, s-IL-6Ra, IL-18, IFN-α, IL-16, PECAM-1 and Prolactin found in patients with CHC as well as with HCC and CHC-related cirrhosis [8–9].

**Table 2:** Statistical evaluation on the serum levels (expressed in pg/mL) of significant cytokines in the healthy controls and in four patient groups belonging to discovery set. We report for each cytokine the minimum and maximum values, the 25% and 75% Percentiles, the median, the mean, standard deviation, standard error, and the lower and upper 95% confidence intervals (CI).

	CTR	T2D	CHC	HCC	T2D-HCC
ADIPOQ					
Minimum (pg/mL)	40200000	40240000	69550000	62550000	66550000
25% Percentile (pg/mL)	45590000	46240000	70240000	63180000	67190000
Median (pg/mL)	60980000	60180000	73180000	73250000	70683331
75% Percentile (pg/mL)	66350000	67700000	80520000	78570000	87570000
Maximum (pg/mL)	66730000	68730000	82670000	87070000	92670000
Mean (pg/mL)	56970000	57610000	74650000	71880000	75158454
Std. Deviation (pg/mL)	11290000	11660000	5661000	9057000	8696033
Std. Error (pg/mL)	5049000	5216000	2831000	3423000	5956000
Lower 95% CI (pg/mL)	42960000	43130000	65640000	63500000	56190000
Upper 95% CI (pg/mL)	70990000	72100000	83650000	80260000	94100000
GLUCAGON					
Minimum (pg/mL)	124.0	453.3	220.0	1088	1400
25% Percentile (pg/mL)	156.3	458.2	235.0	1256	1407
Median (pg/mL)	265.0	923.0	300.0	1390	1629
75% Percentile (pg/mL)	303.3	992.5	450.0	3239	2093
Maximum (pg/mL)	312.0	1026	510.0	6177	2224
Mean (pg/mL)	241.5	764.9	334.0	2437	1650
Std. Deviation (pg/mL)	81.99	282.4	117.6	1837	248.9
Std. Error (pg/mL)	41.00	126.3	52.59	694.3	191.1
Lower 95% CI (pg/mL)	111.0	414.2	188.0	738.0	1079
Upper 95% CI (pg/mL)	372.0	1116	480.0	4136	2296
β-NGF					
Minimum (pg/mL)	0.3100	0.3700	0.8000	1.470	3.770
25% Percentile (pg/mL)	0.3700	0.3700	0.8250	1.655	4.578
Median (pg/mL)	0.6000	0.3700	0.9500	2.610	7.550
75% Percentile (pg/mL)	0.7100	0.6400	1.300	3.735	8.140
Maximum (pg/mL)	0.7300	0.7300	1.400	4.090	8.140
Mean (pg/mL)	0.5600	0.4600	1.025	2.678	6.975
Std. Deviation (pg/mL)	0.1822	0.1800	0.2630	1.079	1.493
Std. Error (pg/mL)	0.09110	0.0900	0.1315	0.4823	1.033
Lower 95% CI (pg/mL)	0.2701	0.1736	0.6065	1.339	3.475
Upper 95% CI (pg/mL)	0.8499	0.7464	1.443	4.017	10.05
CXCL1					
Minimum (pg/mL)	22.52	24.47	50.52	46.10	104.6
25% Percentile (pg/mL)	33.50	31.00	52.91	49.91	105.3
Median (pg/mL)	60.85	62.25	64.65	73.19	200.4
75% Percentile (pg/mL)	63.63	64.93	83.30	95.78	380.8
Maximum (pg/mL)	65.01	65.01	90.74	110.7	424.4
Mean (pg/mL)	51.02	50.82	67.51	72.91	212.5
Std. Deviation (pg/mL)	17.84	18.71	16.48	25.40	95.21
Std. Error (pg/mL)	7.980	8.367	6.729	11.36	75.61
Lower 95% CI (pg/mL)	28.86	27.59	50.22	41.37	-18.97
Upper 95% CI (pg/mL)	73.18	74.05	84.81	104.5	462.3
CXCL12					
Minimum (pg/mL)	20.35	29.35	45.00	71.33	122.0
25% Percentile (pg/mL)	25.18	29.68	47.50	76.93	141.4
Median (pg/mL)	34.00	34.00	61.00	88.00	205.6
75% Percentile (pg/mL)	40.79	45.79	70.00	114.6	242.5
Maximum (pg/mL)	42.58	52.58	71.00	121.5	250.0
Mean (pg/mL)	33.19	36.99	59.50	94.21	202.6
Std. Deviation (pg/mL)	8.626	9.530	11.82	20.15	32.8
Std. Error (pg/mL)	3.858	4.262	5.909	9.010	27.33
Lower 95% CI (pg/mL)	22.48	25.15	40.69	69.19	110.9
Upper 95% CI (pg/mL)	43.90	48.82	78.31	119.2	284.9
CXCL9					
Minimum (pg/mL)	188.4	188.4	1400	2062	1673
25% Percentile (pg/mL)	427.4	230.8	1500	2346	2117
Median (pg/mL)	705.9	666.5	1800	3842	4073
75% Percentile (pg/mL)	1396	1163	1950	6297	5750
Maximum (pg/mL)	1519	1619	2100	8443	6000
Mean (pg/mL)	870.6	690.6	1740	4421	4106
Std. Deviation (pg/mL)	528.4	567.7	260.8	2325	1141.5
Std. Error (pg/mL)	236.3	253.9	116.6	822.0	944.9
Lower 95% CI (pg/mL)	214.5	-14.31	1416	2478	1024
Upper 95% CI (pg/mL)	1527	1396	2064	6365	7038
HGF					
Minimum (pg/mL)	459.1	569.1	450.0	1014	65.92
25% Percentile (pg/mL)	489.6	614.1	480.0	1510	79.78
Median (pg/mL)	619.2	757.3	680.0	1774	162.7
75% Percentile (pg/mL)	658.7	1165	720.0	2456	208.5
Maximum (pg/mL)	659.1	1181	740.0	2831	210.0
Mean (pg/mL)	589.1	863.1	616.0	1900	150.3
Std. Deviation (pg/mL)	94.03	283.8	127.8	591.2	69.32
Std. Error (pg/mL)	47.02	126.9	57.15	187.0	34.66
Lower 95% CI (pg/mL)	439.5	510.8	457.3	1477	40.04
Upper 95% CI (pg/mL)	738.8	1215	774.7	2323	260.6
IFN-α					
Minimum (pg/mL)	3.190	4.190	3.500	28.45	65.92
25% Percentile (pg/mL)	3.393	4.190	4.250	45.08	79.78
Median (pg/mL)	4.095	4.595	7.000	59.34	169.20
75% Percentile (pg/mL)	5.210	5.750	8.500	71.76	208.5
Maximum (pg/mL)	5.550	6.000	9.000	106.1	210.0
Mean (pg/mL)	4.233	4.845	6.500	60.70	161.81
Std. Deviation (pg/mL)	0.9795	0.8595	2.236	22.78	42.38
Std. Error (pg/mL)	0.4898	0.4297	1.000	7.593	34.66
Lower 95% CI (pg/mL)	2.674	3.477	3.724	43.19	40.04
Upper 95% CI (pg/mL)	5.791	6.213	9.276	78.21	260.6
IL-16					
Minimum (pg/mL)	34.36	44.36	150.0	353.8	497.7
25% Percentile (pg/mL)	61.94	66.94	175.0	355.2	579.3
Median (pg/mL)	117.6	117.6	220.0	444.3	942.2
75% Percentile (pg/mL)	142.3	154.8	290.0	522.8	1894
Maximum (pg/mL)	151.7	171.7	330.0	545.7	2192
Mean (pg/mL)	105.2	112.2	230.0	440.1	1015.5
Std. Deviation (pg/mL)	45.67	48.33	66.71	85.39	440.6
Std. Error (pg/mL)	20.43	21.62	29.83	38.19	369.5
Lower 95% CI (pg/mL)	48.49	52.19	147.2	334.1	-47.48
Upper 95% CI (pg/mL)	161.9	172.2	312.8	546.1	2304
IL-18					
Minimum (pg/mL)	22.00	54.86	70.00	161.4	268.9
25% Percentile (pg/mL)	24.75	63.64	79.00	206.7	326.6
Median (pg/mL)	36.50	88.10	105.0	326.4	551.2
75% Percentile (pg/mL)	43.00	113.5	120.0	372.6	694.5
Maximum (pg/mL)	44.00	125.9	130.0	396.7	725.9
Mean (pg/mL)	34.75	88.46	100.6	291.6	532.1
Std. Deviation (pg/mL)	9.639	27.13	22.73	88.09	114.0
Std. Error (pg/mL)	4.820	12.13	10.17	33.29	96.71
Lower 95% CI (pg/mL)	19.41	54.78	72.37	210.2	215.8
Upper 95% CI (pg/mL)	50.09	122.1	128.8	373.1	831.4
IL-2R					
Minimum (pg/mL)	31.00	61.73	75.61	171.3	576.9
25% Percentile (pg/mL)	37.00	67.89	89.83	195.7	682.7
Median (pg/mL)	50.00	137.0	137.0	303.1	1713.2
75% Percentile (pg/mL)	65.00	167.0	175.1	564.5	2939
Maximum (pg/mL)	75.00	175.6	191.7	681.5	3174
Mean (pg/mL)	50.80	121.4	133.4	365.3	1765.1
Std. Deviation (pg/mL)	16.25	50.88	45.39	195.6	708.5
Std. Error (pg/mL)	7.269	22.75	20.30	69.14	591.5
Lower 95% CI (pg/mL)	30.62	58.18	76.99	201.8	-136.6
Upper 95% CI (pg/mL)	70.98	184.5	189.7	528.8	3628
Leptin					
Minimum (pg/mL)	3820	10000	25000	11770	1274
25% Percentile (pg/mL)	4323	23420	30000	13390	1602
Median (pg/mL)	6917	44020	38250	18380	2778.7
75% Percentile (pg/mL)	10150	57910	42500	29120	5270
Maximum (pg/mL)	10870	65820	45000	38250	6000
Mean (pg/mL)	7131	41330	36650	20680	2967.9
Std. Deviation (pg/mL)	3022	20520	7449	10320	1182.5
Std. Error (pg/mL)	1511	9176	3331	4615	997.3
Lower 95% CI (pg/mL)	2323	15860	27400	7866	61.65
Upper 95% CI (pg/mL)	11940	66810	45900	33490	6409
PECAM-1					
Minimum (pg/mL)	16900	168850	30030	22530	30920
25% Percentile (pg/mL)	19600	19170	31760	29290	33190
Median (pg/mL)	26020	22310	33950	32480	43197.6
75% Percentile (pg/mL)	33380	31660	40660	42670	49150
Maximum (pg/mL)	35300	35890	45510	55510	50440
Mean (pg/mL)	26400	24800	35810	35800	42653.7
Std. Deviation (pg/mL)	7280	7081	5680	9966	5018.9
Std. Error (pg/mL)	3256	3167	2319	3322	4167
Lower 95% CI (pg/mL)	17360	16000	29850	28140	28400
Upper 95% CI (pg/mL)	35430	33590	41770	43460	54920
Prolactin					
Minimum (pg/mL)	18140	18260	21930	5655	4550
25% Percentile (pg/mL)	20030	20030	23030	8760	5912
Median (pg/mL)	26560	36560	28760	18530	10914.2
75% Percentile (pg/mL)	38760	43760	35220	24700	21610
Maximum (pg/mL)	41200	46320	36560	29300	24760
Mean (pg/mL)	28830	32830	29000	17510	11859.3
Std. Deviation (pg/mL)	9701	12260	6303	8279	5109.9
Std. Error (pg/mL)	4338	5481	3151	2760	4275
Lower 95% CI (pg/mL)	16780	17610	18970	11140	-740.4
Upper 95% CI (pg/mL)	40870	48050	39030	23870	26470
sIL-6Ra					
Minimum (pg/mL)	35780	45780	55780	77270	160000
25% Percentile (pg/mL)	38060	52100	57300	79320	162500
Median (pg/mL)	47990	71460	66460	98930	177407
75% Percentile (pg/mL)	51650	74130	73940	129800	187700
Maximum (pg/mL)	51840	74900	74900	156700	190000
Mean (pg/mL)	45900	65900	65900	103400	176227
Std. Deviation (pg/mL)	7429	13520	8693	31720	8960.9
Std. Error (pg/mL)	3715	6758	4347	14180	6507
Lower 95% CI (pg/mL)	34080	44390	52070	64050	154500
Upper 95% CI (pg/mL)	57720	87410	79730	142800	195900
VEGFR-1					
Minimum (pg/mL)	500.0	1683	1097	855.8	1056
25% Percentile (pg/mL)	514.1	1846	1220	1159	1087
Median (pg/mL)	617.8	2096	1282	1262	1292
75% Percentile (pg/mL)	799.3	2217	1460	1468	1460
Maximum (pg/mL)	839.4	2251	1492	1705	1500
Mean (pg/mL)	643.8	2044	1305	1288	1288
Std. Deviation (pg/mL)	150.4	221.5	138.8	233.4	124.8
Std. Error (pg/mL)	75.18	99.06	52.47	73.80	96.42
Lower 95% CI (pg/mL)	404.5	1769	1177	1121	961.9
Upper 95% CI (pg/mL)	883.0	2320	1434	1455	1576
VEGFR-2					
Minimum (pg/mL)	5055	15150	9258	9866	9375
25% Percentile (pg/mL)	5291	15870	9866	11660	10030
Median (pg/mL)	6990	19720	11450	13080	12791
75% Percentile (pg/mL)	9027	22260	12580	13730	18540
Maximum (pg/mL)	9375	24750	13530	14750	20060
Mean (pg/mL)	7103	19190	11190	12760	14445
Std. Deviation (pg/mL)	1945	3695	1576	1413	3793.6
Std. Error (pg/mL)	972.3	1652	595.7	446.7	2272
Lower 95% CI (pg/mL)	4008	14610	9730	11750	6621
Upper 95% CI (pg/mL)	10200	23780	12650	13770	21080

**Table 3:** Comparison of cytokine serum levels between patients and healthy controls in the discovery set. We report the results of all the performed statistical analysis obtained by the nonparametric Mann-Whitney U test in terms of U test and P values, by the Unparied t test in terms of P value, t, the number of degrees of freedom (df), the difference between the means, 95% confidence interval, and R squared, and by F test in terms of F, degrees of freedom for the numerator (DFn) and for the denominator (Dfd) and P value. In particular, we reported in bold the values of p<0.05 indicated with *, of p<0.01 with **, and of p<0.0001 with ***.

ADIPOQ	CTR vs T2D	CTR vs CHC	CTR vs HCC	CHC vs HCC	T2D vs HCC	T2D vs T2D-HCC	HCC vs T2D-HCC
*Mann-Whitney Utest*							
U test	11.5	0	6	11	6	2	12
P value	0.44	0.045*	0.038*	0.71	0.035*	0.048*	0.15
*Unpaired t test*							
P value	0.9319	0.0253*	0.0292*	0.5982	0.0377*	0.0418*	0.6181
t, df	t = 0.08816 df = 8	t = 2.832 df = 7	t = 2.543 df = 10	t = 0.5462 df = 9	t = 2.393 df = 10	t = 2.221 df = 7	t = 0.5163 df = 9
Difference between means	-640000 ± 7259000	-17670000 ± 6241000	-14910000 ± 5861000	2768000 ± 5067000	-14270000 ± 5961000	-17530000 ± 7896000	-3268000 ± 6330000
95% confidence interval	-17380000 to 16100000	-32430000 to -2914000	-27960000 to -1847000	-8695000 to 14230000	-27550000 to -985100	-36210000 to 1141000	-17590000 to 11050000
R squared	0.0009707	0.5339	0.3928	0.03209	0.3642	0.4133	0.02876
*F test to compare variances*							
F,DFn, Dfd	1.068, 4, 4	3.976, 4, 3	1.554, 4, 6	2.559, 6, 3	1.659, 4, 6	1.043, 3, 4	1.730, 3, 6
P value	0.9510	0.2860	0.5989	0.4713	0.5517	0.9289	0.5196
**GLUCAGON**							
*Mann-Whitney Utest*							
U test	0	7	0	0	0	0	12
P value	0.041*	0.55	0.011*	0.041*	0.0295*	0.0159*	0.57
*Unpaired t test*							
P value	0.0094**	0.2259	0.0444*	0.0304*	0.0245*	0.0041**	0.4505
t, df	t = 3.544 df = 7	t = 1.328 df = 7	t = 2.334 df = 9	t = 2.521 df = 10	t = 2.991 df = 10	t = 4.181 df = 7	t = 0.7887 df = 9
Difference between means	-523.4 ± 147.7	-92.50 ± 69.66	-2195 ± 940.6	-2103 ± 834.4	-1672 ± 839.7	-922.6 ± 220.7	749.5 ± 950.3
95% confidence interval	-872.6 to -174.2	-257.3 to 72.25	-4323 to -67.82	-3962 to -244.1	-3543 to 198.8	-1444 to -400.8	-1400 to 2899
R squared	0.6422	0.2012	0.3771	0.3885	0.2839	0.7141	0.06465
*F test to compare variances*							
F,DFn, Dfd	11.86, 4, 3	2.057, 4, 3	502.0, 6, 3	244.0, 6, 4	42.32, 6, 4	1.833, 3, 4	23.09, 6, 3
P value	0.0699	0.5798	0.0003***	**P<0.0001**	0.0028**	0.5628	0.0263*
**β-NGF**							
*Mann-Whitney Utest*							
U test	6.5	0	0	0	0	0	1
P value	0.32	0.021*	0.0021**	0.041*	0.0018**	0.0001***	0.010**
*Unpaired t test*							
P value	0.4646	0.0271*	0.0064**	0.0212*	0.0051**	0.0009***	0.0062**
t, df	t = 0.7809 df = 6	t = 2.907 df = 6	t = 3.832 df = 7	t = 2.957 df = 7	t = 4.014 df = 7	t = 6.078 df = 6	t = 3.855 df = 7
Difference between means	0.1000 ± 0.1281	-0.4650 ± 0.1600	-2.118 ± 0.5527	-1.653 ± 0.5590	-2.218 ± 0.5526	-6.303 ± 1.037	-4.085 ± 1.059
95% confidence interval	-0.2134 to 0.4134	-0.8565 to -0.07354	-3.425 to -0.8108	-2.975 to -0.3311	-3.525 to -0.9111	-8.840 to -3.765	-6.590 to -1.579
R squared	0.09225	0.5847	0.6772	0.5554	0.6971	0.8603	0.6798
*F test to compare variances*							
F,DFn, Dfd	1.025, 3, 3	2.083, 3, 3	35.04, 4, 3	16.82, 4, 3	35.90, 4, 3	131.8, 3, 3	3.670, 3, 4
P value	0.9845	0.5620	0.0150*	0.0430*	0.0145*	0.0022**	0.2413
**CXCL1**							
*Mann-Whitney Utest*							
U test	11	1.1	0.9	14.5	0.6	0	1.1
P value	0.88	0.035*	0.039*	0.75	0.041*	0.01**	0.0381*
*Unpaired t test*							
P value	0.9866	0.0415*	0.0453*	0.6800	0.456*	0.0383*	0.0438*
t, df	t = 0.01730 df = 8	t = 1.953 df = 9	t = 1.977 df = 8	t = 0.4261 df = 9	t = 1.966 df = 8	t = 2.547 df = 7	t = 2.199 df = 7
Difference between means	0.2000 ± 11.56	-16.49 ± 10.36	-21.89 ± 13.88	-5.399 ± 12.67	-22.09 ± 14.11	-170.8 ± 67.08	-148.7 ± 67.64
95% confidence interval	-26.46 to 26.86	-39.92 to 6.930	-53.91 to 10.12	-34.06 to 23.26	-54.63 to 10.44	-329.5 to -12.19	-308.7 to 11.24
R squared	0.00003740	0.2199	0.2371	0.01978	0.2346	0.4809	0.4085
*F test to compare variances*							
F,DFn, Dfd	1.099, 4, 4	1.172, 4, 5	2.027, 4, 4	2.375, 4, 5	1.844, 4, 4	65.33, 3, 4	35.44, 3, 4
P value	0.9291	0.8457	0.5107	0.3683	0.5680	0.0015**	0.0049**
**CXCL12**							
*Mann-Whitney Utest*							
U test	11.5	0	0	0	0	0	0
P value	0.76	0.0364*	0.0031**	0.038*	0.0011**	0.0070**	0.0196*
*Unpaired t test*							
P value	0.5272	0.0061**	0.0003***	0.0191*	0.0004***	0.0003***	0.0054**
t, df	t = 0.6611 df = 8	t = 3.877 df = 7	t = 6.226 df = 8	t = 3.029 df = 7	t = 5.741 df = 8	t = 6.570 df = 7	t = 3.974 df = 7
Difference between means	-3.800 ± 5.748	-26.31 ± 6.787	-61.02 ± 9.801	-34.71 ± 11.46	-57.22 ± 9.967	-160.9 ± 24.49	-103.7 ± 26.09
95% confidence interval	-17.06 to 9.456	-42.37 to -10.26	-83.62 to -38.42	-61.81 to -7.606	-80.20 to -34.24	-218.8 to -103.0	-165.4 to -41.98
R squared	0.05179	0.6823	0.8289	0.5672	0.8047	0.8605	0.6929
*F test to compare variances*							
F,DFn, Dfd	1.221, 4, 4	1.877, 3, 4	5.455, 4, 4	2.906, 4, 3	4.469, 4, 4	32.91, 3, 4	7.364, 3, 4
P value	0.8515	0.5488	0.1291	0.4074	0.1761	0.0056**	0.0835
**CXCL9**							
*Mann-Whitney Utest*							
U test	10.5	1	0	1	0	0	14
P value	0.59	0.041*	0.0023**	0.042*	0.0011**	0.0435*	0.16
*Unpaired t test*							
P value	0.6178	0.0109*	0.0070**	0.0281*	0.0052**	0.0067**	0.7781
t, df	t = 0.5189 df = 8	t = 3.299 df = 8	t = 3.310 df = 11	t = 2.527 df = 11	t = 3.470 df = 11	t = 3.803 df = 7	t = 0.2895 df = 10
Difference between means	180.0 ± 346.9	-869.4 ± 263.5	-3551 ± 1073	-2681 ± 1061	-3731 ± 1075	-3340 ± 878.4	390.6 ± 1349
95% confidence interval	-619.9 to 979.9	-1477 to -261.7	-5912 to -1190	-5017 to -345.9	-6097 to -1364	-5418 to -1263	-2616 to 3397
R squared	0.03257	0.5764	0.4990	0.3673	0.5226	0.6738	0.008309
*F test to compare variances*							
F,DFn, Dfd	1.154, 4, 4	4.106, 4, 4	19.36, 7, 4	79.49, 7, 4	16.77, 7, 4	11.08, 3, 4	1.514, 7, 3
P value	0.8927	0.2001	0.0123**	0.0008***	0.0162**	0.0417*	0.7962
**HGF**							
*Mann-Whitney Utest*							
U test	2.5	7	2	0	2	0	5
P value	0.045*	0.89	0.01**	0.0013**	0.027*	0.0016**	0.036*
*Unpaired t test*							
P value	0.0489*	0.7369	0.0010**	0.0004***	0.0029**	0.0038**	0.0109*
t, df	t = 1.990 df = 7	t = 0.3497 df = 7	t = 4.309 df = 12	t = 4.716 df = 13	t = 3.665 df = 13	t = 4.254 df = 7	t = 3.006 df = 12
Difference between means	-274.0 ± 149.7	-26.87 ± 76.84	-1311 ± 304.2	-1284 ± 272.2	-1037 ± 282.9	-2510 ± 590.2	-1474 ± 490.3
95% confidence interval	-628.0 to 80.06	-208.6 to 154.9	-1973 to -647.8	-1872 to -695.8	-1648 to -425.6	-3906 to -1115	-2542 to -405.4
R squared	0.3237	0.01717	0.6074	0.6311	0.5081	0.7210	0.4295
*F test to compare variances*							
F,DFn, Dfd	9.107, 4, 3	1.847, 4, 3	39.53, 9, 3	21.40, 9, 4	4.341, 9, 4	21.10, 3, 4	4.860, 3, 9
P value	0.1001	0.6415	0.0117*	0.0098**	0.1711	0.0130*	0.0562
**IFN-α**							
*Mann-Whitney Utest*							
U test	4	4	0	0	0	0	2
P value	0.72	0.66	0.035*	0.041*	0.0069**	0.0040**	0.0152*
*Unpaired t test*							
P value	0.3835	0.1037	0.0005***	0.0002***	0.0006***	0.0057**	0.0039**
t, df	t = 0.9400 df = 6	t = 1.870 df = 7	t = 4.836 df = 11	t = 5.212 df = 12	t = 4.784 df = 11	t = 4.198 df = 6	t = 3.631 df = 11
Difference between means	-0.6125 ± 0.6516	-2.268 ± 1.213	-56.47 ± 11.68	-54.20 ± 10.40	-55.85 ± 11.68	-145.5 ± 34.66	-89.64 ± 24.69
95% confidence interval	-2.207 to 0.9819	-5.136 to 0.6007	-82.17 to -30.76	-76.86 to -31.54	-81.55 to -30.15	-230.3 to -60.68	-144.0 to -35.31
R squared	0.1284	0.3331	0.6801	0.6936	0.6753	0.7460	0.5452
*F test to compare variances*							
F,DFn, Dfd	1.299, 3, 3	5.211, 4, 3	540.7, 8, 3	103.8, 8, 4	702.4, 8, 3	6505, 3, 3	9.261, 3, 8
P value	0.8349	0.2063	0.0002***	0.0005***	0.0002***	P<0.0001***	0.0111*
**IL-16**							
*Mann-Whitney Utest*							
U test	11	1	0	0	0	0	2
P value	0.89	0.031*	0.004**	0.048*	0.0013**	0.0059**	0.0132*
*Unpaired t test*							
P value	0.8198	0.0087**	**P<0.0001**	0.0467*	0.0346*	0.0168*	0.0436*
t, df	t = 0.2354 df = 8	t = 3.452 df = 8	t = 7.733 df = 8	t = 1.937 df = 12	t = 2.382 df = 12	t = 3.123 df = 7	t = 2.102 df = 7
Difference between means	-7.000 ± 29.74	-124.8 ± 36.16	-334.9 ± 43.31	-516.0 ± 266.5	-633.8 ± 266.0	-1016 ± 325.5	-688.4 ± 327.4
95% confidence interval	-75.58 to 61.58	-208.2 to -41.42	-434.8 to -235.0	-1097 to 64.56	-1214 to -54.13	-1786 to -246.5	-1463 to 85.96
R squared	0.006878	0.5983	0.8820	0.2381	0.3211	0.5821	0.3871
*F test to compare variances*							
F,DFn, Dfd	1.120, 4, 4	2.133, 4, 4	3.495, 4, 4	76.43, 8, 4	145.6, 8, 4	233.8, 3, 4	74.91, 3, 4
P value	0.9153	0.4811	0.2529	0.0008***	0.0002***	0.0001***	0.0011**
**IL-18**							
*Mann-Whitney Utest*							
U test	0	0	0	0	0	0	4
P value	0.041*	0.023*	0.0021**	0.039*	0.030*	0.0001***	0.034*
*Unpaired t test*							
P value	0.0073**	0.0011**	0.0003***	0.0009***	0.0006***	0.0015**	0.0212*
t, df	t = 3.732 df = 7	t = 5.362 df = 7	t = 5.682 df = 9	t = 4.679 df = 10	t = 4.932 df = 10	t = 5.057 df = 7	t = 2.786 df = 9
Difference between means	-53.71 ± 14.39	-65.85 ± 12.28	-256.9 ± 45.21	-191.0 ± 40.83	-203.2 ± 41.20	-435.1 ± 86.04	-232.0 ± 83.25
95% confidence interval	-87.74 to -19.67	-94.89 to -36.81	-359.2 to -154.6	-282.0 to -100.1	-295.0 to -111.4	-638.6 to -231.6	-420.3 to -43.64
R squared	0.6655	0.8042	0.7820	0.6865	0.7087	0.7851	0.4631
*F test to compare variances*							
F,DFn, Dfd	7.919, 4, 3	5.562, 4, 3	83.51, 6, 3	15.01, 6, 4	10.55, 6, 4	50.84, 3, 4	4.821, 3, 6
P value	0.1206	0.1902	0.0040**	0.0205*	0.0391*	0.0024**	0.0973
**IL-2R**							
*Mann-Whitney Utest*							
U test	0	0	0	2	1	0	2
P value	0.049*	0.031*	0.003**	0.036*	0.023*	0.016*	0.0132*
*Unpaired t test*							
P value	0.0183*	0.0050**	0.0047**	0.0261*	0.0210*	0.0168*	0.0071**
t, df	t = 2.954 df = 8	t = 3.829 df = 8	t = 3.529 df = 11	t = 2.568 df = 11	t = 2.691 df = 11	t = 3.123 df = 7	t = 3.374 df = 10
Difference between means	-70.55 ± 23.89	-82.55 ± 21.56	-314.5 ± 89.11	-231.9 ± 90.29	-243.9 ± 90.64	-1625 ± 520.2	-1381 ± 409.3
95% confidence interval	-125.6 to -15.47	-132.3 to -32.83	-510.6 to -118.3	-430.7 to -33.18	-443.4 to -44.42	-2855 to -394.3	-2293 to -468.8
R squared	0.5217	0.6469	0.5310	0.3749	0.3970	0.5822	0.5323
*F test to compare variances*							
F,DFn, Dfd	9.797, 4, 4	7.799, 4, 4	144.8, 7, 4	18.56, 7, 4	14.77, 7, 4	540.7, 3, 4	36.60, 3, 7
P value	0.0483*	0.0716	0.0002***	0.0134*	0.0205*	P<0.0001***	0.0002***
**Leptin**							
*Mann-Whitney Utest*							
U test	1	0	0	2.5	6	0	0
P value	0.011*	0.035*	0.002**	0.041*	0.012*	0.0016**	0.0112*
*Unpaired t test*							
P value	0.0138*	0.022*	0.0404*	0.0017**	0.0059**	0.0082**	0.0133*
t, df	t = 3.261 df = 7	t = 2.373 df = 7	t = 2.510 df = 7	t = 4.031 df = 12	t = 3.335 df = 12	t = 3.649 df = 7	t = 3.288 df = 7
Difference between means	-34200 ± 10490	-29520 ± 4004	-13550 ± 5399	23720 ± 5886	28410 ± 8517	38100 ± 10440	17440 ± 5306
95% confidence interval	-59010 to -9397	-38990 to -20050	-26320 to -780.5	10900 to 36550	9848 to 46970	13400 to 62790	4895 to 29990
R squared	0.6030	0.8859	0.4736	0.5752	0.4811	0.6554	0.6069
*F test to compare variances*							
F,DFn, Dfd	46.11, 4, 3	6.078, 4, 3	11.67, 4, 3	2.510, 8, 4	3.023, 4, 8	105.8, 4, 3	26.77, 4, 3
P value	0.0100*	0.1700	0.0715	0.3903	0.1713	0.0029**	0.0221*
**PECAM-1**							
*Mann-Whitney Utest*							
U test	10.5	4	9	36.5	7	1	11
P value	0.84	0.041*	0.032*	0.77	0.029*	0.0377*	0.33
*Unpaired t test*							
P value	0.7337	0.0390*	0.0405*	0.6774	0.0157*	0.0133*	0.3290
t, df	t = 0.3523 df = 8	t = 2.413 df = 9	t = 1.991 df = 12	t = 0.4233 df = 17	t = 2.702 df = 16	t = 3.290 df = 7	t = 1.021 df = 11
Difference between means	1600 ± 4542	-9410 ± 3900	-9405 ± 5109	-1798 ± 4248	-12810 ± 4741	-16870 ± 5127	-5861 ± 5738
95% confidence interval	-8873 to 12070	-18230 to -589.6	-20540 to 1726	-10760 to 7165	-22860 to -2758	-28990 to -4741	-18490 to 6768
R squared	0.01528	0.3929	0.2203	0.01043	0.3133	0.6072	0.08664
*F test to compare variances*							
F,DFn, Dfd	1.057, 4, 4	1.643, 4, 5	1.874, 8, 4	2.837, 12, 5	1.825, 12, 4	1.385, 3, 4	1.430, 8, 3
P value	0.9585	0.5940	0.5690	0.2581	0.5917	0.7374	0.8433
**Prolactin**							
*Mann-Whitney Utest*							
U test	9.5	9.5	9	4	8	2	12
P value	0.77	0.62	0.041*	0.047*	0.045*	0.043*	0.57
*Unpaired t test*							
P value	0.5829	0.9764	0.0393*	0.0319*	0.0158*	0.0285*	0.3751
t, df	t = 0.5722 df = 8	t = 0.03066 df = 7	t = 2.313 df = 12	t = 2.456 df = 11	t = 2.807 df = 12	t = 2.749 df = 7	t = 0.9244 df = 11
Difference between means	-4000 ± 6990	-173.1 ± 5644	11320 ± 4897	11500 ± 4681	15320 ± 5458	19960 ± 7261	4641 ± 5020
95% confidence interval	-20120 to 12120	-13520 to 13180	654.1 to 21990	1193 to 21800	3430 to 27220	2792 to 37140	-6409 to 15690
R squared	0.03932	0.0001343	0.3083	0.3541	0.3964	0.5192	0.07208
*F test to compare variances*							
F,DFn, Dfd	1.596, 4, 4	2.369, 4, 3	1.373, 4, 8	1.726, 8, 3	2.191, 4, 8	2.055, 4, 3	1.067, 3, 8
P value	0.6616	0.5046	0.6499	0.7106	0.3206	0.5805	0.8317
**sIL-6Ra**							
*Mann-Whitney Utest*							
U test	2	0	0	0	0	0	0
P value	0.032*	0.039*	0.0037**	0.032*	0.021*	0.0001***	0.024*
*Unpaired t test*							
P value	0.0410*	0.0129*	0.0099**	0.0119*	0.0125*	P<0.0001***	0.0040**
t, df	t = 2.594 df = 6	t = 3.498 df = 6	t = 3.505 df = 7	t = 3.010 df = 11	t = 2.981 df = 11	t = 11.65 df = 6	t = 4.205 df = 7
Difference between means	-20000 ± 7711	-20000 ± 5718	-57530 ± 16410	-69420 ± 23060	-69420 ± 23290	-109300 ± 9381	-71760 ± 17070
95% confidence interval	-38870 to -1131	-33990 to -6009	-96340 to -18720	-120200 to -18660	-120700 to -18160	-132200 to -86340	-112100 to -31400
R squared	0.5286	0.6710	0.6371	0.4517	0.4468	0.9577	0.7163
*F test to compare variances*							
F,DFn, Dfd	3.309, 3, 3	1.369, 3, 3	18.22, 4, 3	26.43, 8, 3	10.93, 8, 3	1.078, 3, 3	5.939, 4, 3
P value	0.3519	0.8024	0.0384	0.0212*	0.0746	0.9519	0.1751
**VEGFR-1**							
*Mann-Whitney Utest*							
U test	0	0	0	32.5	1	0	18.5
P value	0.0004***	0.0043**	0.0031**	0.71	0.0013**	0.0016**	0.89
*Unpaired t test*							
P value	P<0.0001***	P<0.0001***	0.0003***	0.8609	0.0011**	0.0009***	0.8882
t, df	t = 10.75 df = 7	t = 7.395 df = 9	t = 5.048 df = 12	t = 0.1782 df = 15	t = 6.012 df = 13	t = 5.515 df = 7	t = 0.1436 df = 12
Difference between means	-1401 ± 130.3	-661.7 ± 89.48	-644.0 ± 127.6	17.65 ± 99.04	756.7 ± 125.9	775.7 ± 140.7	19.03 ± 132.5
95% confidence interval	-1709 to -1093	-864.1 to -459.3	-922.0 to -366.0	-193.4 to 228.7	484.8 to 1029	443.0 to 1108	-269.7 to 307.7
R squared	0.9429	0.8587	0.6799	0.002114	0.7355	0.8129	0.001716
*F test to compare variances*							
F,DFn, Dfd	2.171, 4, 3	1.173, 3, 6	2.410, 9, 3	2.827, 9, 6	1.110, 9, 4	1.319, 4, 3	1.465, 9, 3
P value	0.5504	0.7904	0.5071	0.2188	0.9950	0.8537	0.8307
**VEGFR-2**							
*Mann-Whitney Utest*							
U test	0	1	0	15.5	0	4	18
P value	0.0005***	0.045*	0.039*	0.68	0.0007***	0.036*	0.89
*Unpaired t test*							
P value	0.0006***	0.0041**	0.0010**	0.482	0.0003***	0.042*	0.4866
t, df	t = 5.872 df = 7	t = 3.816 df = 9	t = 2.116 df = 12	t = 2.151 df = 15	t = 4.975 df = 13	t = 1.951 df = 7	t = 0.7179 df = 12
Difference between means	-12090 ± 2059	-4085 ± 1070	-5654 ± 924.5	-1569 ± 729.5	6438 ± 1294	5342 ± 2737	-1096 ± 1527
95% confidence interval	-16960 to -7222	-6506 to -1663	-7668 to -3639	-3124 to -14.66	3643 to 9233	-1132 to 11820	-4423 to 2231
R squared	0.8313	0.6180	0.7571	0.2358	0.6556	0.3523	0.04118
*F test to compare variances*							
F,DFn, Dfd	3.610, 4, 3	1.522, 3, 6	1.895, 3, 9	1.245, 6, 9	6.841, 4, 9	1.513, 3, 4	10.35, 3, 9
P value	0.3199	0.6044	0.4019	0.7365	0.0164	0.6803	0.0056

Comparing the serum levels in CHC and HCC patients we can underline that the concentrations of β-NGF, CXCL9, CXCL12, IL-16, IL-18, IL-2R, Leptin, sIL-6Ra were higher in HCC patients and indicated as possible index of the chronic inflammation leading in CHC patients to the HCC development. Moreover, since the stage of fibrosis in CHC patients has been determined by Ishak index (Table 1), we divided these patients into three subgroups corresponding to stages F2, F3 and F4. No significant difference was observed in CHC patients matching F3 and F4 grades probably because they corresponded to two stages of fibrosis, already well advanced. The comparison of F2 and F4 patients showed that the concentrations of IL-2R, CXCL9, CXCL12 and sIL-6Ra were statistically higher (with p<0.05) in CHC patients with F4 grade. In overall, we find that these results are in agreement with those recently published by our group [15].

Finally, we have also compared the serum levels of these proteins in patients with T2D and HCC evidencing that glucagon, HGF, β-NGF, CXCL1, CXCL9, CXCL12, IFN-α, IL-2R, IL-16, IL-18, PECAM-1 and Prolactin are higher in HCC patients, whereas leptin, sVEGFR-1 and sVEGFR-2 are lower than in patients with T2D. No difference of sIL-6R levels is evident between T2D and HCC patients.

### Comparison between Patients with T2D-HCC and those with T2D or HCC

Since our aim is to identify new markers specific for the association between diabetes and HCC, we compared the levels of all the 49 proteins evaluated in T2D-HCC patients and in those with T2D or HCC alone. From the Fig 1 and the Tables 2 and 3, we can underline that: i) the levels of ADIPOQ, β-NGF, CXCL1, CXCL12, HGF, IL-2R, sIL-6Ra, IL-16, IL-18, IFN-α were higher in T2D-HCC patients in comparison with those with T2D or HCC, ii) the levels of LEP were lower in T2D-HCC patients in comparison with those with T2D or HCC, iii) the levels of CXCL9, PECAM-1, Prolactin, glucagon, sVEGFR-1 and sVEGFR-2 presented similar levels in patients with only HCC and with both T2D and HCC, iv) the levels of CXCL9, PECAM-1, Prolactin, and glucagon were higher in T2D-HCC patients than in those with only T2D, and v) the levels of sVEGFR-1 and sVEGFR-2 were lower in T2D-HCC patients than in those with only T2D.

Then, we have correlated the serum levels of all the significant proteins in T2D-HCC patients with clinical/biochemical data by means of the Pearson correlation coefficient. In these patients, IL-18 showed a significant correlation with alpha-fetoprotein (AFP) and glycemic levels while HGF only with AFP. This suggests that IL-18 can be used as an index of the co-presence of type 2 diabetes and liver cancer whereas HGF is specific only for the cancer. Moreover, CXCL9 and Prolactin resulted to be correlated with the transaminases (AST and ALT), thus confirming that these proteins can be considered as predictors of inflammatory activation during the progression of T2D and HCV-related cirrhosis, which leads to the cancer.

### Functional and network analysis

In general, epidemiological studies show that the liver carcinogenesis has very complex etiologies and, in addition to being associated with viral infection, is also connected with other risk factors such as obesity and T2D. In this context the availability of large amounts of molecular data, as the ones we have collected, can give rise to computational analyses aimed at creating new concepts and statistical and computational models. In this context, it is not easy to correlate those clinical and molecular data, which may be the most representative and sensitive to distinguish the stages of progression of the different syndromes, considered individually. So, to understand in which metabolic pathways all the major proteins that we identified were involved, we conducted a functional analysis using the DAVID tool [13]. This analysis was also supplemented by a network study with the Ingenuity Pathway Analysis (IPA). **Table 4** shows how these seventeen proteins, based on what is known in literature, may be involved in six metabolic pathways.

**Table 4:** Metabolic pathways showing the constitutive proteins considered as significantly involved.

Pathways	Proteins
Cytokine-cytokine receptor interaction	CXCL1, CXCL9, CXCL12, HGF, IL-2RA, IL-6R, IL-18, LEP, PRL, VEGFR-1, VEGFR-2
JAK-STAT signaling	IL-2RA, IL-6R, LEP, PRL
Hepatic fibrosis/Hepatic Stellate Cell Activation	CXCL9, HGF, IL-6R, LEP, VEGFR-1, VEGFR-2
Granulocyte Adhesion and Diapedesis	CXCL1, CXCL9, CXCL12, IL-18, PECAM-1
NFkB signaling	IL-18, NGF, VEGFR-1, VEGFR-2
Role of macrophages, fibroblasts, and endothelial cells	CXCL12, IL-6R, IL-16, IL-18

**Fig 2:**
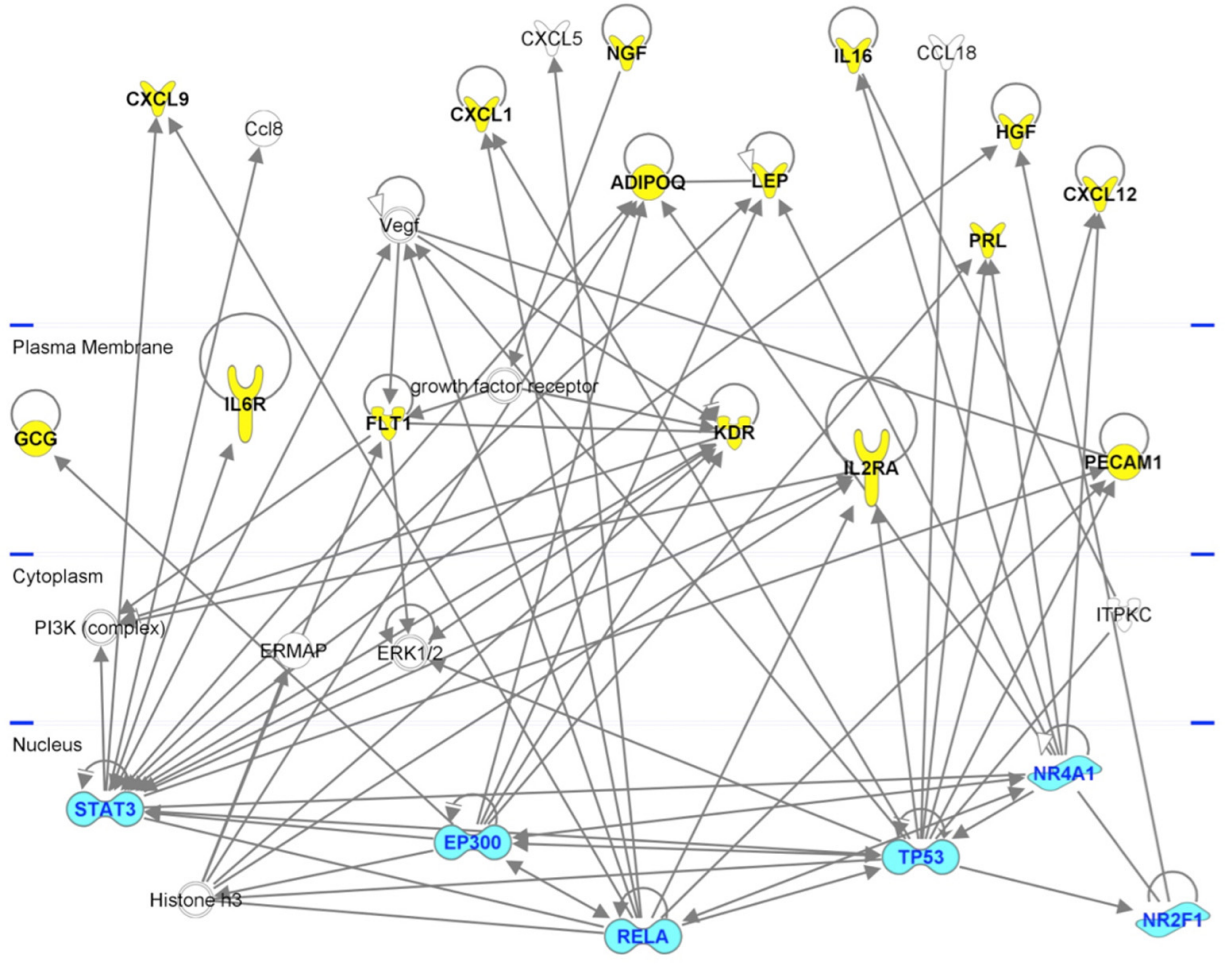
Interactomic analysis of the significant molecules performed by means of the Ingenuity Pathway Analysis (IPA). The interactome shows the close functional association between significant cytokines (evidenced by yellow symbols) as well as the paths in which other functionally relevant molecules are also involved (evidenced by white symbols). Moreover, the six HUB nodes are evidenced by cyan symbols. On the left side the cellular localization of the molecules in the graph is shown.

The interactomic analysis shows that all the significant cytokines are involved in a network named “*Cellular movement*, *Hematological System Development and Function*, *Immune Cell Trafficking*” on the basis of the function associated with them and of data mining from the experimental studies reported in the literature (**Fig 2**). This network reveals that these proteins are connected by six HUB nodes, such as EP300 (E1A binding protein p300), NR4A1 (nuclear receptor subfamily 4, group A, member 1), NR2F1 (nuclear receptor subfamily 2, group F, member 1), RELA (nuclear factor NF-kappa-B p65 subunit), STAT3 (signal-transducer-and-activator-of-transcription 3) and TP53 (tumor protein p53), which are closely related between them. The hub nodes, representing the centers of metabolic correlation that exercise a direct control over the coordinated proteins and often through the formation of a complex, have a strategic value, both because they centralize the control and because they are the best targets for each project aimed at creating specific drugs. In details we can underline that: i) EP300 is connected with ADIPOQ, Glucagon (GCG), sVEGFR-2 (KDR), Leptin (LEP), and Prolactin (PRL), ii) NR2F1 with HGF, iii) NR4A1 with ADIPOQ, CXCL12, IL-16, Leptin (LEP), and Prolactin (PRL), iv) RELA with CXCL9, CXCL12, IL-2RA, PECAM-1, and VEGF that interacts with its two receptors, VEGFR-1 (FLT1) and VEGFR-2 (KDR), v) STAT3 with ADIPOQ, CXL9, HGF, IL-2RA, sVEGFR-2 (KDR), Leptin (LEP), and PECAM-1, and vi) TP53 with CXCL1, CXCL12, IL-2RA, PECAM-1, Prolactin, and VEGF as in the case of RELA.

To experimentally verify the putative interactions found between our significant cytokines and the HUB nodes by means of the network analysis, we have determined the serum concentrations of the TP53 protein in the patients with HCC and T2D-HCC. As shown in **S1 Table**, 19 patients with HCC and 6 with T2D-HCC resulted negative to TP53 antibody while 15 patients with HCC and 4 with T2D-HCC were positive. In addition, we have also correlated the concentrations of TP53 with those of CXCL1, CXCL12, IL-2RA, PECAM-1, and Prolactin because they have been found associated with TP53 from the network analysis. TP53 showed no correlation with CXCL1, IL-2RA, PECAM-1, and Prolactin whereas a significant correlation (with p-values <0.05) has been found with CXCL12 in HCC as well as in T2D-HCC patients.

### Bio-Plex Assay on validation set

**Fig 3:**
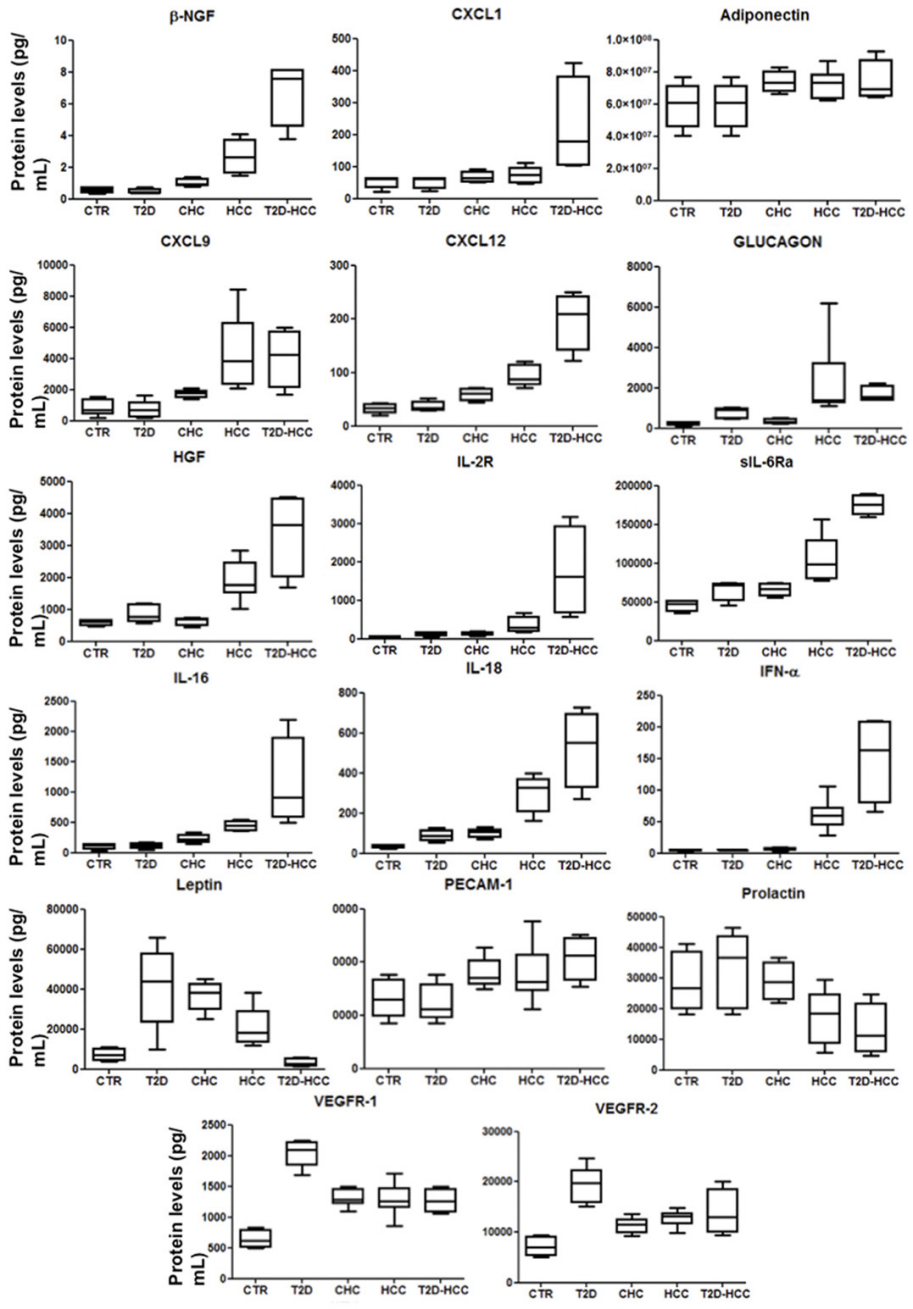
Significant cytokines in some patient groups belonging to validation set. We report the significant molecule levels from controls, patients with type 2 diabetes (T2D), chronic hepatitis C (CHC), hepatocellular carcinoma (HCC) and hepatocellular carcinoma and type 2 diabetes (T2D-HCC) shown by means of box-and-whisker graphs. The boxes extend from the 25th to the 75th percentile, and the line in the middle is the median. The error bars extend down to the lowest value and up to the highest.

To validate all the results we have evaluated the serum levels of cytokines, growth factors, chemokines, as well as of other cancer and diabetes biomarkers in a validation set, including 20 patients with T2D, 20 patients with CHC, 20 with HCC, 10 with T2D-HCC, and 20 healthy control subjects (Fig 3 and S2 Table). Then, we compared the obtained serum levels between the patients and healthy controls by the Mann Whitney U-test, the Unparied t test and F test and obtained that the same proteins, already resulted in the discovery set, were significant also in the patients groups belonging to the validation set (S3 Table).

Furthermore, we compared also the serum levels for the significant proteins obtained for this validation cohort with those evaluated in the discovery set. As shown in Table 5, we have verified that all the P values were higher than 0.05 and, hence, no statistically significant difference exists between the two subject groups (“discovery set” and “validation set”). This evidences the reliability of our results, and the possibility to use them for discriminating the different patient groups.

**Table 5:** Comparison of cytokine serum levels between discovery and validation sets in patients and healthy controls. We report the results of all the performed statistical analysis obtained by the nonparametric Mann-Whitney U test in terms of U test and P values, by the Unparied t test in terms of P value, t, the number of degrees of freedom (df), the difference between the means, 95% confidence interval, and R squared, and by F test in terms of F, degrees of freedom for the numerator (DFn) and for the denominator (Dfd) and P value.

	CTR vs CTR^V^	T2D vs T2D^V^	CHC vs CHC^V^	HCC vs HCC^V^	T2D-HCC vs T2D-HCC^V^
**ADIPOQ**					
*Mann-Whitney Utest*					
U-test	12.5	11	8.5	23.5	6.5
P-value	0.9166	0.8413	0.8057	0.949	0.7715
*Unpaired t test*					
P value	0.7829	0.7086	0.8682	1.0000	0.5762
t, df	t = 0.2850 df = 8	t = 0.3874 df = 8	t = 0.1722 df = 7	t = 0.0000 df = 12	t = 0.5909 df = 6
Difference between means	-1888000 ± 6623000	-2480000 ± 6402000	933200 ± 5420000	0.0000 ± 4178000	-4861000 ± 8227000
95% confidence interval	-17160000 to 13390000	-17240000 to 12280000	-11880000 to 13750000	-9103000 to 9103000	-24990000 to 15270000
R squared	0.01005	0.01841	0.004218	0.0000	0.05499
*F test to compare variances*					
F,DFn, Dfd	1.386, 4, 4	1.975, 4, 4	2.814, 4, 3	2.043, 6, 6	1.101, 3, 3
P value	0.7592	0.5260	0.4217	0.4059	0.9386
**GLUCAGON**					
*Mann-Whitney Utest*					
U-test	5.5	10	11	22.5	5.5
P-value	0.5614	0.6905	0.8335	0.8478	0.5614
*Unpaired t test*					
P value	0.4629	0.8907	0.7513	0.6242	0.6009
t, df	t = 0.7840 df = 6	t = 0.1419 df = 8	t = 0.3280 df = 8	t = 0.5027 df = 12	t = 0.5519 df = 6
Difference between means	-41.25 ± 52.62	-24.52 ± 172.8	-25.40 ± 77.44	376.4 ± 748.8	-199.8 ± 361.9
95% confidence interval	-170.0 to 87.50	-423.0 to 373.9	-204.0 to 153.2	-1255 to 2008	-1085 to 685.8
R squared	0.09292	0.002511	0.01327	0.02063	0.04832
*F test to compare variances*					
F,DFn, Dfd	1.545, 3, 3	1.147, 4, 4	1.168, 4, 4	6.139, 6, 6	2.585, 3, 3
P value	0.7294	0.8974	0.8839	0.0441	0.4560
**β-NGF**					
*Mann-Whitney Utest*					
U-test	7	7.5	6.5	10.5	10
P-value	0.8857	1	0.7715	0.753	0.9009
*Unpaired t test*					
P value	0.8544	0.8710	0.4273	0.8926	0.8335
t, df	t = 0.1915 df = 6	t = 0.1695 df = 6	t = 0.8513 df = 6	t = 0.1394 df = 8	t = 0.2182 df = 7
Difference between means	-0.02500 ± 0.1305	-0.02250 ± 0.1328	-0.2375 ± 0.2790	-0.08800 ± 0.6312	0.3105 ± 1.423
95% confidence interval	-0.3444 to 0.2944	-0.3473 to 0.3023	-0.9202 to 0.4452	-1.544 to 1.368	-3.054 to 3.675
R squared	0.006075	0.004765	0.1078	0.002424	0.006758
*F test to compare variances*					
F,DFn, Dfd	1.053, 3, 3	1.176, 3, 3	3.501, 3, 3	1.403, 4, 4	1.094, 4, 3
P value	0.9670	0.8973	0.3308	0.7508	0.9804
**CXCL1**					
*Mann-Whitney Utest*					
U-test	11	10.5	17	15	10
P-value	0.8335	0.7533	0.6161	0.7436	0.9017
*Unpaired t test*					
P value	0.5960	0.7636	0.8059	0.7940	0.9462
t, df	t = 0.5520 df = 8	t = 0.3112 df = 8	t = 0.2518 df = 11	t = 0.2683 df = 10	t = 0.06993 df = 7
Difference between means	-5.574 ± 10.10	-3.814 ± 12.26	-2.150 ± 8.538	3.846 ± 14.34	-6.450 ± 92.23
95% confidence interval	-28.86 to 17.71	-32.08 to 24.45	-20.94 to 16.64	-28.10 to 35.79	-224.6 to 211.7
R squared	0.03669	0.01196	0.005729	0.007145	0.0006981
*F test to compare variances*					
F,DFn, Dfd	1.663, 4, 4	1.146, 4, 4	1.323, 5, 6	1.134, 4, 6	1.435, 3, 4
P value	0.6342	0.8983	0.7344	0.8465	0.7142
**CXCL12**					
*Mann-Whitney Utest*					
U-test	12.5	11.5	7	11	7
P-value	0.9161	0.916	0.883	0.8335	0.8839
*Unpaired t test*					
P value	0.9604	0.8203	0.7749	0.8126	0.8651
t, df	t = 0.05122 df = 8	t = 0.2348 df = 8	t = 0.2991 df = 6	t = 0.2450 df = 8	t = 0.1773 df = 6
Difference between means	-0.3040 ± 5.936	1.374 ± 5.852	-2.738 ± 9.151	-3.382 ± 13.80	6.730 ± 37.96
95% confidence interval	-13.99 to 13.38	-12.12 to 14.87	-25.13 to 19.66	-35.21 to 28.44	-86.16 to 99.62
R squared	0.0003278	0.006844	0.01469	0.007450	0.005211
*F test to compare variances*					
F,DFn, Dfd	1.367, 4, 4	1.130, 4, 4	1.399, 3, 3	1.347, 4, 4	1.077, 3, 3
P value	0.7690	0.9088	0.7894	0.7801	0.9529
**CXCL9**					
*Mann-Whitney Utest*					
U-test	11.5	10	11	30	7
P-value	0.9166	0.674	0.834	0.8745	0.8857
*Unpaired t test*					
P value	0.9226	0.8130	0.9399	0.8860	0.8300
t, df	t = 0.1003 df = 8	t = 0.2446 df = 8	t = 0.07785 df = 8	t = 0.1459 df = 14	t = 0.2242 df = 6
Difference between means	-34.34 ± 342.5	-85.41 ± 349.2	14.07 ± 180.8	149.2 ± 1022	-302.3 ± 1348
95% confidence interval	-824.2 to 755.5	-890.7 to 719.9	-402.8 to 431.0	-2044 to 2342	-3601 to 2997
R squared	0.001255	0.007420	0.0007570	0.001519	0.008309
*F test to compare variances*					
F,DFn, Dfd	1.101, 4, 4	1.121, 4, 4	1.403, 4, 4	1.829, 7, 7	1.036, 3, 3
P value	0.9281	0.9144	0.7508	0.4440	0.9776
**HGF**					
*Mann-Whitney Utest*					
U-test	7	11	13.5	49	9.5
P-value	0.8857	0.8335	0.569	0.9698	1
*Unpaired t test*					
P value	0.9206	0.9427	0.5776	0.8967	0.8874
t, df	t = 0.1039 df = 6	t = 0.07416 df = 8	t = 0.5756 df = 10	t = 0.1317 df = 18	t = 0.1469 df = 7
Difference between means	7.380 ± 71.01	-13.33 ± 179.7	44.51 ± 77.33	33.27 ± 252.6	113.0 ± 769.1
95% confidence interval	-166.4 to 181.1	-427.8 to 401.2	-127.8 to 216.8	-497.4 to 564.0	-1706 to 1932
R squared	0.001797	0.0006870	0.03207	0.0009631	0.003073
*F test to compare variances*					
F,DFn, Dfd	1.281, 3, 3	1.006, 4, 4	1.113, 6, 4	1.211, 9, 9	1.655, 3, 4
P value	0.8435	0.9954	0.9608	0.7797	0.6239
**IFN-α**					
*Mann-Whitney Utest*					
U-test	8	7	11.5	40	7.5
P value	1	0.8846	0.9166	0.708	1
*Unpaired t test*					
P value	0.7778	0.7616	0.8668	0.7240	0.6738
t, df	t = 0.2952 df = 6	t = 0.3175 df = 6	t = 0.1732 df = 8	t = 0.3590 df = 17	t = 0.4423 df = 6
Difference between means	-0.2625 ± 0.8892	-0.2225 ± 0.7007	0.2240 ± 1.294	3.109 ± 8.660	-18.77 ± 42.42
95% confidence interval	-2.438 to 1.913	-1.937 to 1.492	-2.759 to 3.207	-15.16 to 21.38	-122.6 to 85.05
R squared	0.01432	0.01653	0.003734	0.007524	0.03158
*F test to compare variances*					
F,DFn, Dfd	2.297, 3, 3	1.659, 3, 3	1.485, 4, 4	2.473, 8, 9	2.007, 3, 3
P value	0.5125	0.6878	0.7111	0.1992	0.5817
**IL-16**					
*Mann-Whitney Utest*					
U-test	10.5	12.5	11.5	7	10.5
P-value	0.753	0.916	0.916	0.9095	0.8307
*Unpaired t test*					
P value	0.7320	0.9652	0.9012	0.3622	0.6969
t, df	t = 0.3547 df = 8	t = 0.04500 df = 8	t = 0.1281 df = 8	t = 0.9662 df = 8	t = 0.4038 df = 8
Difference between means	-10.68 ± 30.12	1.378 ± 30.63	5.136 ± 40.08	-53.80 ± 55.68	142.5 ± 352.9
95% confidence interval	-80.15 to 58.78	-69.24 to 72.00	-87.30 to 97.57	-182.2 to 74.60	-671.3 to 956.4
R squared	0.01548	0.0002530	0.002048	0.1045	0.01998
*F test to compare variances*					
F,DFn, Dfd	1.175, 4, 4	1.007, 4, 4	1.242, 4, 4	1.126, 4, 4	3.626, 3, 5
P value	0.8797	0.9944	0.8388	0.9113	0.1994
**IL-18**					
*Mann-Whitney Utest*					
U-test	7	12	9	17.5	9
P-value	0.8857	1	0.576	0.6678	0.6095
*Unpaired t test*					
P value	0.8273	0.7391	0.4978	0.6730	0.7028
t, df	t = 0.2279 df = 6	t = 0.3448 df = 8	t = 0.7101 df = 8	t = 0.4335 df = 11	t = 0.3955 df = 8
Difference between means	2.123 ± 9.312	-7.288 ± 21.14	12.01 ± 16.91	19.34 ± 44.62	40.02 ± 101.2
95% confidence interval	-20.66 to 24.91	-56.03 to 41.45	-26.99 to 51.00	-78.86 to 117.5	-193.3 to 273.4
R squared	0.008585	0.01464	0.05930	0.01680	0.01918
*F test to compare variances*					
F,DFn, Dfd	2.733, 3, 3	2.036, 4, 4	1.766, 4, 4	1.604, 6, 5	2.216, 3, 5
P value	0.4309	0.5081	0.5951	0.6207	0.4087
**IL-2R**					
*Mann-Whitney Utest*					
U-test	7	9	9	25.5	11.5
P-value	0.3095	0.5476	0.5476	0.5283	1
*Unpaired t test*					
P value	0.1398	0.6507	0.4295	0.7792	0.9263
t, df	t = 1.639 df = 8	t = 0.4703 df = 8	t = 0.8321 df = 8	t = 0.2858 df = 14	t = 0.09544 df = 8
Difference between means	-16.95 ± 10.34	-17.21 ± 36.60	-29.05 ± 34.91	-23.12 ± 80.90	55.71 ± 583.7
95% confidence interval	-40.79 to 6.893	-101.6 to 67.20	-109.5 to 51.45	-196.7 to 150.4	-1290 to 1402
R squared	0.2515	0.02690	0.07965	0.005800	0.001137
*F test to compare variances*					
F,DFn, Dfd	1.023, 4, 4	1.588, 4, 4	1.957, 4, 4	2.709, 7, 7	2.987, 3, 5
P value	0.9828	0.6650	0.5314	0.2119	0.2695
**Leptin**					
*Mann-Whitney Utest*					
U-test	12	9	14	9	5
P-value	1	0.5476	0.9307	0.5476	0.8571
*Unpaired t test*					
P value	0.9622	0.3256	0.6686	0.7404	0.5868
t, df	t = 0.04894 df = 8	t = 1.047 df = 8	t = 0.4424 df = 9	t = 0.3431 df = 8	t = 0.5804 df = 5
Difference between means	80.00 ± 1635	-10500 ± 10020	-3057 ± 6910	-1818 ± 5298	-831.8 ± 1433
95% confidence interval	-3690 to 3850	-33610 to 12620	-18690 to 12570	-14040 to 10400	-4516 to 2853
R squared	0.0002993	0.1206	0.02128	0.01450	0.06312
*F test to compare variances*					
F,DFn, Dfd	1.908, 3, 5	5.182, 4, 4	3.425, 5, 4	3.146, 4, 4	1.403, 3, 2
P value	0.4927	0.1401	0.2565	0.2929	0.8836
**PECAM-1**					
*Mann-Whitney Utest*					
U-test	9	10	8	10	7
P-value	0.9048	1	0.4762	0.2601	0.8857
*Unpaired t test*					
P value	0.8756	0.9450	0.4509	0.3406	0.6969
t, df	t = 0.1624 df = 7	t = 0.07152 df = 7	t = 0.7926 df = 8	t = 0.9962 df = 11	t = 0.4088 df = 6
Difference between means	823.6 ± 5072	-391.1 ± 5468	-3212 ± 4053	-5560 ± 5581	-2317 ± 5669
95% confidence interval	-11170 to 12820	-13320 to 12540	-12560 to 6134	-17850 to 6725	-16190 to 11550
R squared	0.003752	0.0007301	0.07280	0.08276	0.02709
*F test to compare variances*					
F,DFn, Dfd	1.184, 3, 4	1.759, 3, 4	1.593, 3, 5	1.931, 8, 3	1.175, 3, 3
P value	0.8424	0.5871	0.6048	0.6369	0.8975
**Prolactin**					
*Mann-Whitney Utest*					
U-test	10	8	6	15	9
P-value	1	0.7302	0.6857	0.7105	0.9048
*Unpaired t test*					
P value	0.9318	0.7506	0.7559	0.7391	0.6458
t, df	t = 0.08866 df = 7	t = 0.3307 df = 7	t = 0.3255 df = 6	t = 0.3416 df = 11	t = 0.4801 df = 7
Difference between means	552.7 ± 6235	2335 ± 7063	-1538 ± 4724	1524 ± 4461	-2299 ± 4789
95% confidence interval	-14190 to 15300	-14370 to 19040	-13100 to 10020	-8294 to 11340	-13630 to 9028
R squared	0.001122	0.01538	0.01735	0.01050	0.03187
*F test to compare variances*					
F,DFn, Dfd	1.237, 4, 3	2.574, 4, 3	1.247, 3, 3	3.565, 8, 3	2.127, 3, 4
P value	0.8970	0.4633	0.8603	0.3237	0.4790
**sIL-6Ra**					
*Mann-Whitney Utest*					
U-test	6	7	6	12	5
P-value	0.648	0.556	0.6857	1	0.1091
*Unpaired t test*					
P value	0.6318	0.5560	0.7160	0.8730	0.1084
t, df	t = 0.5046 df = 6	t = 0.6181 df = 7	t = 0.3815 df = 6	t = 0.1651 df = 8	t = 1.782 df = 9
Difference between means	2888 ± 5723	4804 ± 7772	-2557 ± 6704	2837 ± 17180	39530 ± 22180
95% confidence interval	-11120 to 16890	-13580 to 23190	-18960 to 13850	-36790 to 42460	-10640 to 89690
R squared	0.04071	0.05176	0.02368	0.003395	0.2609
*F test to compare variances*					
F,DFn, Dfd	1.373, 3, 3	1.865, 3, 4	1.379, 3, 3	2.138, 4, 4	10.59, 6, 3
P value	0.8005	0.5525	0.7979	0.4798	0.0793
**VEGFR-1**					
*Mann-Whitney Utest*					
U-test	7	12	7	15	7
P-value	0.8857	1	0.2303	0.5395	0.8857
*Unpaired t test*					
P value	0.6152	0.9469	0.1429	0.4231	0.6452
t, df	t = 0.5299 df = 6	t = 0.06876 df = 8	t = 1.605 df = 9	t = 0.8293 df = 12	t = 0.4845 df = 6
Difference between means	-50.67 ± 95.63	11.83 ± 172.1	143.8 ± 89.57	110.3 ± 133.0	-74.32 ± 153.4
95% confidence interval	-284.7 to 183.3	-384.9 to 408.6	-58.83 to 346.4	-179.5 to 400.1	-449.7 to 301.1
R squared	0.04470	0.0005907	0.2226	0.05421	0.03765
*F test to compare variances*					
F,DFn, Dfd	1.618, 3, 3	2.017, 4, 4	1.180, 3, 6	1.405, 9, 3	1.531, 3, 3
P value	0.7023	0.5135	0.7863	0.8620	0.7348
**VEGFR-2**					
*Mann-Whitney Utest*					
U-test	7	6	9	25	6
P-value	0.8857	0.4127	0.4121	1	0.6857
*Unpaired t test*					
P value	0.6770	0.5581	0.4362	0.9312	0.5172
t, df	t = 0.4376 df = 6	t = 0.6148 df = 7	t = 0.8148 df = 9	t = 0.08796 df = 13	t = 0.6880 df = 6
Difference between means	483.4 ± 1105	-1604 ± 2608	-860.8 ± 1056	71.61 ± 814.1	1672 ± 2430
95% confidence interval	-2220 to 3187	-7772 to 4565	-3251 to 1529	-1687 to 1830	-4274 to 7618
R squared	0.03093	0.05124	0.06870	0.0005948	0.07312
*F test to compare variances*					
F,DFn, Dfd	3.435, 3, 3	1.251, 3, 4	1.431, 3, 6	1.348, 4, 9	6.965, 3, 3
P value	0.3379	0.8053	0.6472	0.6495	0.1452

## Discussion

In this paper we report a simultaneous and comparative analysis of the serum levels of a large panel of cytokines, growth factors, chemokines, as well as of other cancer and diabetes biomarkers in patients with T2D, CHC, HCC and T2D-HCC by means of BioPlex assays. Our interest for these diseases depends from the fact that Southern Italy shows a high mortality trend for liver cancer in CHC patients [16] concomitantly with very high rates of T2D [17]. Recently we evaluated the cytokinome profile in patients with T2D and/or CHC infection or with CHC-related HCC suggesting some specific markers for the different stages of the diseases [8–9, 15, 18]. Since both T2D and CHC have been identified as contributory causes of HCC [2], our aim is to identify new possible diagnostic/prognostic markers useful for recognizing the features of the association between T2D and HCC.

A general view of the results in Table 3 shows that IL-2R, IL-18, Leptin, sIL-6Ra, sVEGFR-1 and sVEGFR-2 are up-expressed in all the patient groups, suggesting that these proteins are involved in those chronic inflammation processes leading to T2D through the metabolic syndrome and, often concomitantly, to cancer, and in particular, IL-18 has shown a significant correlation with AFP and glycemic levels. On the other hand, we have to underline that the presence of some of these proteins is also due to the necro-inflammatory activity of the liver. Indeed, IL-2R and sIL-6Ra show a significant correlation with the fibrotic stage of our CHC patients, as well the elevated levels of leptin indicate that immune response and host defense, active during infection and inflammation, are acting as paracrine modulator of the hepatic fibrogenesis [19].

It is also interesting to note that ADIPOQ, β-NGF, CXCL1, CXCL9, CXCL12, IL-16, and PECAM-1 are up-expressed only in those CHC and HCC patients who present a liver failure (Table 3), and, hence, linkable to the necro-inflammatory activity of the liver. In fact, it is known that in CHC patients ADIPOQ was found related to the severity of the fibrosis and suggested as HCC marker when the carcinogenesis is concomitantly supported by CHC infection [20] while β-NGF and IL-16 are involved in cancer growth and metastasis and also detected in diseased liver tissues [9, 21]. Similarly, CXCL1 and CXCL9 have chemotactic activities and roles in angiogenesis, inflammation and tumor genesis [9], CXCL12 is related to the HCC metastatic network by recruiting endothelial cell tumor progenitors [22], and PECAM-1 reflects the liver disease progression [23]. However, we have also found that CXCL9 and CXCL12 resulted statistically higher in CHC patients with F4 grade in respect to those with F2 grade, thus confirming their important role in the liver necro-inflammation.

In Table 3 we also show that the levels of HGF and glucagon were higher in T2D and HCC patients but not in those with CHC, suggesting that these two proteins can be related to the pro-inflammatory condition. In details, elevated HGF levels suggest atherosclerotic complications in T2D patients [9] whereas those of glucagon confirm its role in the dysregulated hepatic glucose production, which is characteristic of the abnormal glucose homeostasis of these patients [24]. However, also in our previous studies [8, 15, 18], HGF was found significantly up-regulated in HCC patients but not in patients with CHC and always correlated with AFP, thus supporting our proposal that this growth factor could be used as an index of cellular growth and of HCC development in patients with chronic inflammation.

Moreover, since IFN-α is a lymphokine with a wide range of biological effects and found up-expressed in pre-operative samples of HCC patients [25] while Prolactin is commonly attributed to an impaired hepatic metabolism of estrogens and associated to liver cirrhosis [8], the fact that we have found both up-expressed only in patients with HCC and that Prolactin results to correlate with the transaminase levels, leads us to think that Prolactin might be used as a severity index of liver disease.

Other points to discuss are the serum levels of β-NGF, CXCL1, CXCL12, HGF, IFN-α, IL-16, IL-18, IL-2R, Leptin and sIL-6Ra found for the T2D-HCC patients. These levels were found to be higher than those of patients with only T2D or HCC suggesting that these proteins are concomitantly involved in both diseases. On the other hand the serum levels of ADIPOQ, CXCL9, PECAM-1, Prolactin, sVEGFR-1 and sVEGFR-2 in the T2D-HCC patients were higher than those of patients with only T2D while they were similar to those of HCC patients, confirming that these proteins are specific for the cancer presence.

We have also attempted to understand how these proteins could be correlated between them on the basis of their known metabolic functions and of all the experimental data reported in the literature.

To this end, we have performed an interactomic analysis which calculated how these proteins are significantly connected in a common metabolic network where they are modulated through six HUB nodes, such as EP300, NR4A1, NR2F1, RELA, STAT3 and TP53. In detail, the transcriptional cofactor, EP300, is involved in several biological phenomena, such as cell proliferation, differentiation and apoptosis; it functions as a pleiotropic coactivator and regulates p53-dependent transcription [26]. It was demonstrated that the levels of EP300 protein expression in HCCs were strongly associated with vascular invasion, intrahepatic metastasis and poor prognosis of HCC patients [26]. In fact, the evaluation of EP300 expression was proposed in a new prognostic model based on high EP300 expression, AFP levels and vascular invasion [27]. Moreover, recently, some authors showed that high glucose levels increased the activity of the transcriptional EP300, which increases TGF-β activity via Smad2 acetylation. However its activation increases both the transactivation of glucagon gene by PAX2A protein [28] and the transcription of leptin gene by p42 C/EBP alpha protein [29]. Moreover, EP300 binds the promoter fragment containing a E2F binding site from human VEGFR-2 gene [30] and the DNA endogenous promoter from the human prolactin gene [31]. These data highlighted the importance of the role played by EP300 in both T2D and HCC and its correlation with ADIPOQ, glucagon, sVEGFR2, Leptin and Prolactin.

Moreover, NR4A1 and NR2F1 are soluble nuclear hormone receptors that regulate liver development, differentiation and function, and are implicated in the modulation of the hepatocyte priming and proliferation in regenerating liver, chronic hepatitis and HCC development. All the early changes essential for the liver regeneration, such as the activation of transcription factors (NF-kB and STAT3), as well as the increased levels of cytokines and growth factors (HGF), can be modulated by members of the NRs superfamily [32]. However, an ever-growing body of evidence suggests that members of this family of nuclear receptors (NRs) could play a pivotal role in glucose homeostasis and the development of T2D [33]. In fact, in fasting mouse, a mutant mouse Nur77 [NR4A1] gene resulted to modulate the expression of ADIPOQ, Leptin, IL-16, Prolactin and CXCL12.

RELA is the major component of NF- κB is activated constitutively in human HCC, and plays a key role in controlling apoptosis in HCC cells, suggesting that the RELA may be an important targets for novel therapeutic approaches in the treatment of the human HCC [34–35]. Recent studies have highlighted the role of NF-kB in the pathogenesis of the insulin resistance and T2D as an independent risk factor for the development of the HCC. In particular, the malignant transformation of the hepatocytes may occur through a pathway of an increased liver cell turnover induced by the chronic liver injury and regeneration in a context of inflammation, immune response and oxidative DNA damage. In fact, RELA is involved in the expression of CXCL1, CXCL9, and IL-2RA [36–37], as well increases the transactivation of a DNA endogenous promoter through the PECAM-1 gene [38]. STAT3 is implicated in the signal transduction by different cytokines, growth factors and oncogenes, and plays an important role in tumorigenesis and, in particular, in HCC through the up-regulation of genes involved in anti-apoptosis, proliferation and angiogenesis [39]. Moreover, its signal-pathway was suggested as a therapeutic target for the T2D and drug discovery [40]. In general, STAT3 decreases the expression of CXCL9 [41] and PECAM-1 [42] and is activated by HGF [43], sIL-6R [44] and Leptin [45] whereas ADIPOQ induces its decreasing [46]. Finally, TP53 is a tumor suppressor that initiates cell-cycle arrest, apoptosis, and senescence in response to the cellular stress to maintain the integrity of the genome. Single base substitutions in TP53 occur in approximately 25% of HCC, suggesting a relevant role for TP53 in this cancer [47]. Moreover, it has been found that an excessive calorie intake led to the accumulation of oxidative stress in the adipose tissue of mice with T2D–like disease and promoted the increased expression of TP53 with an associated increased production of pro-inflammatory cytokines [48]. In particular, it is reported that the mutant TP53 increases the expression of CXCL1 [49] and decreases that of PECAM-1 [50], PRL [51] and IL-2RA [52], and its inactivation decreases expression of CXCL12 that involves oncogenic mutant HRAS protein [53]. To support the conclusion of the metabolic network analysis, we have experimentally evaluated the serum levels of TP53 in HCC and T2D patients, showing how they are correlated with the CXCL12 levels and thus confirming that TP53 can suppress the production of this chemokine as already reported in cultured fibroblasts of both human and mouse origin [54]. However, this result confirm the predictive validity of the interactomic analysis because the data that are used for the calculation of the metabolic networks are based exclusively on experimental results from which we can extract complex relationships, not easily detectable but made perceptible through the representation of a mathematical graph that visually illustrates the metabolic net.

## Conclusions

What appears evident from the analysis of our data is that the HCC itself is a disease with a very complex and multifactorial etiology. When associated with other syndromes, the difficulty of being able to follow the onset of the cancer and its progression in time, becomes much more difficult and often elusive. In fact, it is well known that the onset of this cancer is often clinically silent, therefore, when the cancer is discovered it is already too late, and this often determines a poor prognosis. For this reason many laboratories seek direct or indirect biomarkers that can be validly correlated with HCC progression and prognosis. What is clear from our results, is the lack of individual markers, suitable to follow validly this cancer because of the clear and extensive metabolic correlations existing between the molecular species, which generate and control it. We think that only through a large, simple and reliable omics approach, such as the cytokinome analysis, supplemented by the common biochemical/clinical data, we can take into account the correlations between the different molecular partners generating a framework that allows us to make a metabolic acceptable prognosis of the various stages of the disease progression. This is particularly important when we have the superposition of chronic liver disease and T2D. Our approach has also been validated by the interactomic analysis that has clearly shown how our results correlate well with the overall picture so far experimentally known for metabolic associations that exist for a complex and multifactorial disease as HCC. In addition, our data also suggest that the best strategy to design new drugs against HCC must have as a specific target the HUB molecules, that is, those metabolic nodes that coordinate and control the functions of many other metabolically relevant molecules.

## Supporting Information

S1 Table Distribution of TP53 antibody in HCC and T2D-HCC patients. In details, we report the number of patients resulted negative or positive to TP53 antibody and the p-values determined between TP53 levels and those of CXCL1, CXCL12, IL-2RA, PECAM1, and PRL using the Pearson correlation coefficient. The statistically significant p-values are reported in bold and underlined.
(DOC)

S2 Table Statistical evaluation on the serum levels (expressed in pg/mL) of significant cytokines in the healthy controls and in four patient groups belonging to validation set. We report for each cytokine the minimum and maximum values, the 25% and 75% Percentiles, the median, the mean, standard deviation, standard error, and the lower and upper 95% confidence intervals (CI).
(DOC)

S3 Table Comparison of cytokine serum levels between patients and healthy controls in the validation set. We report the results of all the performed statistical analysis obtained by the nonparametric Mann-Whitney U test in terms of U test and P values, by the Unparied t test in terms of P value, t, the number of degrees of freedom (df), the difference between the means, 95% confidence interval, and R squared, and by F test in terms of F, degrees of freedom for the numerator (DFn) and for the denominator (Dfd) and P value. In particular, we reported in bold the values of p<0.05 indicated with *, of p<0.01 with **, and of p<0.0001 with ***.
(DOC)
