# Supplementary material for: The Cytokinome Profile in Patients with Hepatocellular Carcinoma and Type 2 Diabetes
Source: PLoS One. 2015 Jul 30;10(7):e0134594. doi: 10.1371/journal.pone.0134594 (PMC4520685; doi:10.1371/journal.pone.0134594)
Supplement: S1 Table — In details, we report the number of patients resulted negative or positive to TP53 antibody and the p-values determined between TP53 levels and those of CXCL1, CXCL12, IL-2RA, PECAM1, and PRL using the Pearson correlation coefficient. The statistically significant p-values are reported in bold and underlined. (DOC) [file pone.0134594.s001.doc]

**S1Table**. Distribution of TP53 antibody in HCC and T2D-HCC patients. In details, we report the number of patients resulted negative or positive to TP53 antibody and the p-values determined between TP53 levels and those of CXCL1, CXCL12, IL-2RA, PECAM1, and PRL using the Pearson correlation coefficient. The statistically significant p-values are reported in bold and underlined.

|  | **TP53**  **Negative Positive** | **p-value (TP53vsCXCL1)** | **p-value (TP53vsCXCL12)** | **p-value (TP53vsIL-2RA)** | **p-value (TP53vsPECAM1)** | **p-value (TP53vsPRL)** |
| --- | --- | --- | --- | --- | --- | --- |
| **HCC** | 19 15 | 0.91 | **0.021*** | 0.88 | 0.59 | 0.12 |
| **T2D-HCC** | 6 4 | 0.88 | **0.034*** | 0.73 | 0.42 | 0.46 |
