# Supplementary material for: The Cytokinome Profile in Patients with Hepatocellular Carcinoma and Type 2 Diabetes
Source: PLoS One. 2015 Jul 30;10(7):e0134594. doi: 10.1371/journal.pone.0134594 (PMC4520685; doi:10.1371/journal.pone.0134594)
Supplement: S2 Table — We report for each cytokine the minimum and maximum values, the 25% and 75% Percentiles, the median, the mean, standard deviation, standard error, and the lower and upper 95% confidence intervals (CI). (DOC) [file pone.0134594.s002.doc]

**S2 Table. Statistical evaluation on the serum levels (expressed in pg/mL) of significant cytokines in the healthy controls and in four patient groups belonging to validation set.** We report for each cytokine the minimum and maximum values, the 25% and 75% Percentiles, the median, the mean, standard deviation, standard error, and the lower and upper 95% confidence intervals (CI).

|  | **CTR** | **T2D** | **CHC** | **HCC** | **T2D-HCC** |
| --- | --- | --- | --- | --- | --- |
| **ADIPOQ** |  |  |  |  |  |
| Minimum (pg/mL) | 43400000 | 47200000 | 64080000 | 63650000 | 65140000 |
| 25% Percentile (pg/mL) | 51630000 | 52960000 | 66130000 | 67070000 | 68840000 |
| Median (pg/mL) | 60190000 | 62280000 | 72470000 | 72550000 | 80556241 |
| 75% Percentile (pg/mL) | 65430000 | 66130000 | 85330000 | 74890000 | 90070000 |
| Maximum (pg/mL) | 69870000 | 69980000 | 89550000 | 83180000 | 92670000 |
| Mean (pg/mL) | 58860000 | 60090000 | 74650000 | 71880000 | 80182817 |
| Std. Deviation (pg/mL) | 9587000 | 8300000 | 10700000 | 6336000 | 6683465 |
| Std. Error | 4288000 | 3712000 | 5349000 | 2395000 | 5676000 |
| Lower 95% CI (pg/mL) | 46960000 | 49790000 | 57620000 | 66020000 | 61950000 |
| Upper 95% CI (pg/mL) | 70770000 | 70400000 | 91670000 | 77740000 | 98070000 |
| **GLUCAGON** |  |  |  |  |  |
| Minimum (pg/mL) | 199.0 | 503.0 | 249.0 | 1088 | 1512 |
| 25% Percentile (pg/mL) | 216.0 | 523.2 | 249.5 | 1406 | 1518 |
| Median (pg/mL) | 289.5 | 823.0 | 350.0 | 1909 | 1643 |
| 75% Percentile (pg/mL) | 342.8 | 1039 | 474.0 | 2503 | 2525 |
| Maximum (pg/mL) | 353.0 | 1110 | 558.0 | 3277 | 2801 |
| Mean (pg/mL) | 282.8 | 789.4 | 359.4 | 2061 | 1743.2 |
| Std. Deviation (pg/mL) | 65.96 | 263.7 | 127.1 | 741.4 | 378.4 |
| Std. Error | 32.98 | 117.9 | 56.84 | 280.2 | 307.3 |
| Lower 95% CI (pg/mL) | 177.8 | 462.0 | 201.6 | 1375 | 909.3 |
| Upper 95% CI (pg/mL) | 387.7 | 1117 | 517.2 | 2746 | 2865 |
| **β-NGF** |  |  |  |  |  |
| Minimum (pg/mL) | 0.3500 | 0.3100 | 0.8600 | 1.880 | 3.500 |
| 25% Percentile (pg/mL) | 0.3975 | 0.3250 | 0.8700 | 2.443 | 4.200 |
| Median (pg/mL) | 0.6000 | 0.4350 | 1.140 | 3.700 | 6.975 |
| 75% Percentile (pg/mL) | 0.7575 | 0.6875 | 1.778 | 7.473 | 8.305 |
| Maximum (pg/mL) | 0.7900 | 0.7500 | 1.910 | 8.470 | 8.470 |
| Mean (pg/mL) | 0.5850 | 0.4825 | 1.263 | 4.609 | 6.607 |
| Std. Deviation (pg/mL) | 0.1870 | 0.1952 | 0.4921 | 2.494 | 1.481 |
| Std. Error | 0.09350 | 0.09759 | 0.2460 | 0.7886 | 0.9665 |
| Lower 95% CI (pg/mL) | 0.2874 | 0.1719 | 0.4795 | 2.825 | 3.769 |
| Upper 95% CI (pg/mL) | 0.8826 | 0.7931 | 2.046 | 6.393 | 9.135 |
| **CXCL1** |  |  |  |  |  |
| Minimum (pg/mL) | 34.25 | 29.68 | 55.71 | 45.90 | 94.41 |
| 25% Percentile (pg/mL) | 44.36 | 33.60 | 58.71 | 46.10 | 101.0 |
| Median (pg/mL) | 60.85 | 61.85 | 65.00 | 66.00 | 250.8 |
| 75% Percentile (pg/mL) | 66.70 | 72.06 | 80.82 | 87.82 | 344.2 |
| Maximum (pg/mL) | 71.15 | 75.87 | 95.11 | 110.7 | 388.4 |
| Mean (pg/mL) | 56.59 | 54.63 | 69.66 | 69.07 | 234.5 |
| Std. Deviation (pg/mL) | 13.84 | 20.03 | 14.33 | 23.86 | 87.8 |
| Std. Error | 6.188 | 8.956 | 5.417 | 9.017 | 56.45 |
| Lower 95% CI (pg/mL) | 39.41 | 29.77 | 56.41 | 47.00 | 71.38 |
| Upper 95% CI (pg/mL) | 73.77 | 79.50 | 82.92 | 91.13 | 384.8 |
| **CXCL12** |  |  |  |  |  |
| Minimum (pg/mL) | 18.79 | 25.94 | 47.54 | 74.52 | 114.5 |
| 25% Percentile (pg/mL) | 24.40 | 27.97 | 49.41 | 86.27 | 135.8 |
| Median (pg/mL) | 34.00 | 34.00 | 61.00 | 114.5 | 201.7 |
| 75% Percentile (pg/mL) | 42.33 | 44.06 | 76.31 | 209.8 | 227.9 |
| Maximum (pg/mL) | 45.66 | 49.12 | 79.41 | 230.6 | 230.6 |
| Mean (pg/mL) | 33.49 | 35.61 | 62.24 | 139.2 | 195.3 |
| Std. Deviation (pg/mL) | 10.09 | 8.966 | 13.98 | 61.21 | 30.65 |
| Std. Error | 4.511 | 4.010 | 6.988 | 20.40 | 26.34 |
| Lower 95% CI (pg/mL) | 20.97 | 24.48 | 40.00 | 92.13 | 107.3 |
| Upper 95% CI (pg/mL) | 46.01 | 46.75 | 84.48 | 186.2 | 275.0 |
| **CXCL9** |  |  |  |  |  |
| Minimum (pg/mL) | 200.4 | 188.4 | 1430 | 2092 | 1953 |
| 25% Percentile (pg/mL) | 413.3 | 330.8 | 1495 | 2732 | 2362 |
| Median (pg/mL) | 805.9 | 776.3 | 1630 | 3873 | 4634 |
| 75% Percentile (pg/mL) | 1446 | 1221 | 2005 | 6101 | 5898 |
| Maximum (pg/mL) | 1619 | 1619 | 2230 | 6392 | 5900 |
| Mean (pg/mL) | 905.0 | 776.0 | 1726 | 4272 | 4461 |
| Std. Deviation (pg/mL) | 554.4 | 536.2 | 308.9 | 1719 | 1156.5 |
| Std. Error | 247.9 | 239.8 | 138.1 | 607.7 | 961.7 |
| Lower 95% CI (pg/mL) | 216.6 | 110.3 | 1342 | 2835 | 1273 |
| Upper 95% CI (pg/mL) | 1593 | 1442 | 2109 | 5709 | 7394 |
| **HGF** |  |  |  |  |  |
| Minimum (pg/mL) | 445.1 | 589.2 | 411.5 | 1121 | 1827 |
| 25% Percentile (pg/mL) | 472.7 | 632.1 | 430.0 | 1485 | 2414 |
| Median (pg/mL) | 596.5 | 757.3 | 547.6 | 1774 | 3232 |
| 75% Percentile (pg/mL) | 676.1 | 1180 | 707.9 | 2406 | 4100 |
| Maximum (pg/mL) | 689.0 | 1212 | 732.8 | 2691 | 4635 |
| Mean (pg/mL) | 581.8 | 876.5 | 571.5 | 1867 | 3263 |
| Std. Deviation (pg/mL) | 106.4 | 284.6 | 134.8 | 537.1 | 690.9 |
| Std. Error | 53.21 | 127.3 | 50.96 | 169.9 | 453.0 |
| Lower 95% CI (pg/mL) | 412.4 | 523.0 | 446.8 | 1482 | 2003 |
| Upper 95% CI (pg/mL) | 751.1 | 1230 | 696.2 | 2251 | 4518 |
| **IFN-a** |  |  |  |  |  |
| Minimum (pg/mL) | 2.980 | 3.760 | 3.900 | 39.40 | 101.3 |
| 25% Percentile (pg/mL) | 3.233 | 3.983 | 4.450 | 48.02 | 117.5 |
| Median (pg/mL) | 4.245 | 5.095 | 6.650 | 56.40 | 175.9 |
| 75% Percentile (pg/mL) | 6.008 | 6.125 | 7.915 | 59.93 | 207.3 |
| Maximum (pg/mL) | 6.510 | 6.320 | 8.500 | 93.60 | 210.0 |
| Mean (pg/mL) | 4.495 | 5.068 | 6.276 | 57.59 | 173.9 |
| Std. Deviation (pg/mL) | 1.484 | 1.107 | 1.835 | 14.48 | 29.58 |
| Std. Error | 0.7422 | 0.5535 | 0.8207 | 4.580 | 24.47 |
| Lower 95% CI (pg/mL) | 2.133 | 3.306 | 3.997 | 47.23 | 91.25 |
| Upper 95% CI (pg/mL) | 6.857 | 6.829 | 8.555 | 67.95 | 247.0 |
| **IL-16** |  |  |  |  |  |
| Minimum (pg/mL) | 50.28 | 45.96 | 159.2 | 366.3 | 488.5 |
| 25% Percentile (pg/mL) | 69.90 | 61.70 | 179.4 | 411.5 | 684.6 |
| Median (pg/mL) | 123.6 | 126.2 | 220.0 | 490.4 | 874.7 |
| 75% Percentile (pg/mL) | 158.0 | 152.3 | 272.7 | 578.0 | 1413 |
| Maximum (pg/mL) | 182.5 | 166.8 | 321.6 | 600.4 | 1491 |
| Mean (pg/mL) | 115.9 | 110.8 | 224.9 | 493.9 | 937.1 |
| Std. Deviation (pg/mL) | 49.50 | 48.51 | 59.86 | 90.60 | 297.4 |
| Std. Error | 22.14 | 21.70 | 26.77 | 40.52 | 158.4 |
| Lower 95% CI (pg/mL) | 54.42 | 50.59 | 150.5 | 381.4 | 578.7 |
| Upper 95% CI (pg/mL) | 177.4 | 171.1 | 299.2 | 606.4 | 1393 |
| **IL-18** |  |  |  |  |  |
| Minimum (pg/mL) | 14.67 | 49.76 | 55.65 | 188.4 | 325.6 |
| 25% Percentile (pg/mL) | 17.33 | 58.15 | 64.44 | 196.7 | 383.3 |
| Median (pg/mL) | 32.42 | 95.32 | 84.21 | 286.7 | 461.5 |
| 75% Percentile (pg/mL) | 48.14 | 133.6 | 114.9 | 333.0 | 591.3 |
| Maximum (pg/mL) | 51.01 | 142.6 | 136.4 | 342.9 | 699.3 |
| Mean (pg/mL) | 32.63 | 95.75 | 88.59 | 272.3 | 476.6 |
| Std. Deviation (pg/mL) | 15.93 | 38.70 | 30.21 | 69.56 | 97.89 |
| Std. Error | 7.967 | 17.31 | 13.51 | 28.40 | 53.04 |
| Lower 95% CI (pg/mL) | 7.272 | 47.69 | 51.08 | 199.3 | 347.2 |
| Upper 95% CI (pg/mL) | 57.98 | 143.8 | 126.1 | 345.3 | 619.9 |
| **IL-2R** |  |  |  |  |  |
| Minimum (pg/mL) | 49.85 | 84.60 | 92.70 | 274.3 | 621.0 |
| 25% Percentile (pg/mL) | 52.23 | 86.95 | 106.0 | 295.4 | 1146 |
| Median (pg/mL) | 69.40 | 103.9 | 153.0 | 338.7 | 1741.6 |
| 75% Percentile (pg/mL) | 82.45 | 207.5 | 223.5 | 528.3 | 2300 |
| Maximum (pg/mL) | 91.20 | 221.5 | 254.0 | 559.2 | 2501 |
| Mean (pg/mL) | 67.75 | 138.6 | 162.4 | 388.4 | 1840.9 |
| Std. Deviation (pg/mL) | 16.44 | 64.12 | 63.50 | 118.8 | 514.2 |
| Std. Error | 7.353 | 28.67 | 28.40 | 42.01 | 279.5 |
| Lower 95% CI (pg/mL) | 47.33 | 58.96 | 83.55 | 289.1 | 971.8 |
| Upper 95% CI (pg/mL) | 88.17 | 218.2 | 241.3 | 487.7 | 2409 |
| **Leptin** |  |  |  |  |  |
| Minimum (pg/mL) | 4239 | 42330 | 24540 | 14300 | 2543 |
| 25% Percentile (pg/mL) | 5225 | 43140 | 26550 | 17090 | 2543 |
| Median (pg/mL) | 6699 | 52980 | 32910 | 23450 | 3782 |
| 75% Percentile (pg/mL) | 9552 | 59940 | 37970 | 27430 | 5875 |
| Maximum (pg/mL) | 9568 | 64400 | 39540 | 29860 | 5875 |
| Mean (pg/mL) | 7051 | 51830 | 32480 | 22500 | 3911.8 |
| Std. Deviation (pg/mL) | 2188 | 9013 | 6150 | 5819 | 814.8 |
| Std. Error | 893.1 | 4031 | 3075 | 2602 | 972.1 |
| Lower 95% CI (pg/mL) | 4755 | 40640 | 22690 | 15270 | -115.2 |
| Upper 95% CI (pg/mL) | 9347 | 63020 | 42260 | 29720 | 8250 |
| **PECAM-1** |  |  |  |  |  |
| Minimum (pg/mL) | 15440 | 14400 | 31270 | 32970 | 35220 |
| 25% Percentile (pg/mL) | 17450 | 16030 | 32420 | 34220 | 36380 |
| Median (pg/mL) | 26680 | 25360 | 38410 | 41990 | 44598 |
| 75% Percentile (pg/mL) | 32590 | 34170 | 46230 | 47880 | 50700 |
| Maximum (pg/mL) | 33500 | 35630 | 47980 | 48510 | 50990 |
| Mean (pg/mL) | 25570 | 25190 | 39020 | 41360 | 43841 |
| Std. Deviation (pg/mL) | 7920 | 9391 | 7168 | 7172 | 4531.9 |
| Std. Error | 3960 | 4695 | 3584 | 3586 | 3844 |
| Lower 95% CI (pg/mL) | 12970 | 10240 | 27610 | 29950 | 31750 |
| Upper 95% CI (pg/mL) | 38180 | 40130 | 50420 | 52770 | 56210 |
| **Prolactin** |  |  |  |  |  |
| Minimum (pg/mL) | 18140 | 21450 | 23170 | 10650 | 9054 |
| 25% Percentile (pg/mL) | 22800 | 29120 | 28730 | 11860 | 9521 |
| Median (pg/mL) | 32970 | 36940 | 37540 | 15960 | 15087 |
| 75% Percentile (pg/mL) | 40540 | 47360 | 47360 | 20130 | 21040 |
| Maximum (pg/mL) | 48510 | 50990 | 50990 | 21350 | 22460 |
| Mean (pg/mL) | 32520 | 37240 | 37260 | 15980 | 15173.8 |
| Std. Deviation (pg/mL) | 10050 | 10110 | 9908 | 4385 | 4013.4 |
| Std. Error | 2787 | 3576 | 3503 | 2192 | 2622 |
| Lower 95% CI (pg/mL) | 26440 | 28780 | 28980 | 9005 | 7885 |
| Upper 95% CI (pg/mL) | 38590 | 45690 | 45540 | 22960 | 22440 |
| **sIL-6Ra** |  |  |  |  |  |
| Minimum (pg/mL) | 33500 | 49880 | 54330 | 69270 | 69270 |
| 25% Percentile (pg/mL) | 34890 | 51380 | 57540 | 84100 | 102900 |
| Median (pg/mL) | 42440 | 60760 | 67600 | 101400 | 175963 |
| 75% Percentile (pg/mL) | 51710 | 70990 | 75950 | 116700 | 176500 |
| Maximum (pg/mL) | 53680 | 72100 | 75990 | 130500 | 188600 |
| Mean (pg/mL) | 43010 | 61100 | 66920 | 100600 | 170008 |
| Std. Deviation (pg/mL) | 8707 | 9896 | 9489 | 21690 | 20121 |
| Std. Error | 4353 | 4426 | 4243 | 9700 | 16000 |
| Lower 95% CI (pg/mL) | 29160 | 48810 | 55140 | 73660 | 96500 |
| Upper 95% CI (pg/mL) | 56870 | 73380 | 78700 | 127500 | 174800 |
| **VEGFR-1** |  |  |  |  |  |
| Minimum (pg/mL) | 534.6 | 1565 | 984.5 | 999.6 | 1033 |
| 25% Percentile (pg/mL) | 570.6 | 1731 | 1013 | 1006 | 1096 |
| Median (pg/mL) | 721.4 | 2099 | 1167 | 1156 | 1389 |
| 75% Percentile (pg/mL) | 791.4 | 2302 | 1305 | 1371 | 1548 |
| Maximum (pg/mL) | 800.4 | 2369 | 1328 | 1399 | 1570 |
| Mean (pg/mL) | 694.4 | 2033 | 1162 | 1177 | 1368 |
| Std. Deviation (pg/mL) | 118.2 | 314.6 | 150.8 | 196.9 | 140.7 |
| Std. Error | 59.10 | 140.7 | 75.39 | 98.47 | 119.3 |
| Lower 95% CI (pg/mL) | 506.3 | 1642 | 921.7 | 864.1 | 963.4 |
| Upper 95% CI (pg/mL) | 882.5 | 2423 | 1402 | 1491 | 1723 |
| **VEGFR-2** |  |  |  |  |  |
| Minimum (pg/mL) | 5130 | 15990 | 9568 | 10290 | 9872 |
| 25% Percentile (pg/mL) | 5536 | 16960 | 10120 | 11140 | 10400 |
| Median (pg/mL) | 6889 | 20610 | 12320 | 12990 | 12008 |
| 75% Percentile (pg/mL) | 7433 | 24830 | 13710 | 14080 | 13650 |
| Maximum (pg/mL) | 7569 | 25990 | 13990 | 14600 | 13880 |
| Mean (pg/mL) | 6619 | 20800 | 12050 | 12680 | 12082 |
| Std. Deviation (pg/mL) | 1049 | 4132 | 1885 | 1640 | 1000 |
| Std. Error | 524.6 | 2066 | 942.7 | 733.5 | 861.1 |
| Lower 95% CI (pg/mL) | 4950 | 14220 | 9048 | 10650 | 9440 |
| Upper 95% CI (pg/mL) | 8289 | 27370 | 15050 | 14720 | 14920 |
