# Supplementary material for: The Cytokinome Profile in Patients with Hepatocellular Carcinoma and Type 2 Diabetes
Source: PLoS One. 2015 Jul 30;10(7):e0134594. doi: 10.1371/journal.pone.0134594 (PMC4520685; doi:10.1371/journal.pone.0134594)
Supplement: S3 Table — We report the results of all the performed statistical analysis obtained by the nonparametric Mann-Whitney U test in terms of U test and P values, by the Unparied t test in terms of P value, t, the number of degrees of freedom (df), the difference between the means, 95% confidence interval, and R squared, and by F test in terms of F, degrees of freedom for the numerator (DFn) and for the denominator (Dfd) and P value. In particular, we reported in bold the values of p<0.05 indicated with *, of p<0.01 with **, and of p<0.0001 with ***. (DOC) [file pone.0134594.s003.doc]

**S3 Table. Comparison of cytokine serum levels between patients and healthy controls in the validation set.** We report the results of all the performed statistical analysis obtained by the nonparametric Mann-Whitney U test in terms of U test and P values, by the Unparied t test in terms of P value, t, the number of degrees of freedom (df), the difference between the means, 95% confidence interval, and R squared, and by F test in terms of F, degrees of freedom for the numerator (DFn) and for the denominator (Dfd) and P value. In particular, we reported in bold the values of p<0.05 indicated with *, of p<0.01 with **, and of p<0.0001 with ***.

| **ADIPOQ** | **CTR vs T2D** | **CTR vs CHC** | **CTR vs HCC** | **CHC vs HCC** | **T2D vs HCC** | **T2D vs T2D-HCC** | **HCC vs T2D-HCC** |
| --- | --- | --- | --- | --- | --- | --- | --- |
| *Mann-Whitney Utest* |  |  |  |  |  |  |  |
| U test | 10 | 1 | 3 | 13 | 3 | 1 | 8 |
| P-value | 0.69 | **0.0317*** | **0.0177*** | 0.927 | **0.0228*** | **0.0366*** | 0.3152 |
| *Unpaired t test* |  |  |  |  |  |  |  |
| P value | 0.8334 | **0.0422*** | **0.0173*** | 0.5970 | **0.0188*** | **0.0185*** | 0.1548 |
| t, df | t=0.2173 df=8 | t=2.335 df=7 | t=2.850 df=10 | t=0.5481 df=9 | t=2.801 df=10 | t=3.053 df=7 | t=1.553 df=9 |
| Difference between means | -1232000 ± 5671000 | -15790000 ± 6761000 | -13020000 ± 4568000 | 2768000 ± 5050000 | -11790000 ± 4208000 | -19910000 ± 6524000 | -8129000 ± 5233000 |
| 95% confidence interval | -14310000 to 11840000 | -31770000 to 203000 | -23200000 to -2841000 | -8655000 to 14190000 | -21160000 to -2411000 | -35340000 to -4486000 | -19970000 to 3709000 |
| R squared | 0.005869 | 0.4378 | 0.4482 | 0.03230 | 0.4396 | 0.5710 | 0.2114 |
| *F test to compare variances* |  |  |  |  |  |  |  |
| F,DFn, Dfd | 1.334, 4, 4 | 1.245, 3, 4 | 2.289, 4, 6 | 2.851, 3, 6 | 1.716, 4, 6 | 1.871, 3, 4 | 3.209, 3, 6 |
| P value | 0.7866 | 0.8083 | 0.3489 | 0.2545 | 0.5279 | 0.5508 | 0.2086 |
| **GLUCAGON** |  |  |  |  |  |  |  |
| *Mann-Whitney Utest* |  |  |  |  |  |  |  |
| U test | 0 | 7 | 0 | 0 | 1 | 0 | 12 |
| P-value | **0.0159*** | 0.556 | **0.0061**** | **0.0025**** | **0.0051**** | **0.0159*** | 0.787 |
| *Unpaired t test* |  |  |  |  |  |  |  |
| P value | **0.0076**** | 0.3140 | **0.0012**** | **0.0005***** | **0.0046**** | **0.0082**** | 0.7027 |
| t, df | t=3.703 df=7 | t=1.085 df=7 | t=4.676 df=9 | t=5.010 df=10 | t=3.630 df=10 | t=3.645 df=7 | t=0.3941 df=9 |
| Difference between means | -506.6 ± 136.8 | -76.65 ± 70.66 | -1778 ± 380.2 | -1701 ± 339.6 | -1271 ± 350.2 | -1098 ± 301.2 | 173.3 ± 439.8 |
| 95% confidence interval | -830.2 to -183.1 | -243.8 to 90.47 | -2638 to -917.8 | -2458 to -944.6 | -2051 to -491.0 | -1810 to -385.5 | -821.5 to 1168 |
| R squared | 0.6621 | 0.1439 | 0.7084 | 0.7151 | 0.5686 | 0.6549 | 0.01696 |
| *F test to compare variances* |  |  |  |  |  |  |  |
| F,DFn, Dfd | 15.98, 4, 3 | 3.713, 4, 3 | 126.3, 6, 3 | 34.03, 6, 4 | 7.907, 6, 4 | 5.433, 3, 4 | 1.455, 6, 3 |
| P value | 0.0462* | 0.3097 | 0.0022** | 0.0043** | 0.0653 | 0.1357 | 0.8160 |
| **β-NGF** |  |  |  |  |  |  |  |
| *Mann-Whitney Utest* |  |  |  |  |  |  |  |
| U test | 5 | 0 | 0 | 1 | 0 | 0 | 1.5 |
| P-value | 0.485 | **0.0286*** | **0.0159*** | **0.0040**** | **0.0020**** | **0.0159*** | **0.0278*** |
| *Unpaired t test* |  |  |  |  |  |  |  |
| P value | 0.4769 | **0.0421*** | **0.0023**** | **0.0231*** | **0.0073**** | **0.010**** | **0.0079**** |
| t, df | t=0.7584 df=6 | t=2.574 df=6 | t=4.651 df=7 | t=2.603 df=12 | t=3.227 df=12 | t=3.431 df=7 | t=3.515 df=8 |
| Difference between means | 0.1025 ± 0.1351 | -0.6775 ± 0.2632 | -2.181 ± 0.4690 | -3.347 ± 1.286 | -4.127 ± 1.279 | -5.970 ± 1.099 | -3.686 ± 1.049 |
| 95% confidence interval | -0.2282 to 0.4332 | -1.322 to -0.03342 | -3.290 to -1.072 | -6.148 to -0.5446 | -6.913 to -1.340 | -8.569 to -3.370 | -6.104 to -1.268 |
| R squared | 0.08748 | 0.5248 | 0.7555 | 0.3608 | 0.4645 | 0.8082 | 0.6069 |
| *F test to compare variances* |  |  |  |  |  |  |  |
| F,DFn, Dfd | 1.089, 3, 3 | 6.925, 3, 3 | 23.71, 4, 3 | 25.68, 9, 3 | 163.2, 9, 3 | 122.6, 4, 3 | 5.633, 4, 4 |
| P value | 0.9456 | 0.1463 | 0.0264* | 0.0219* | 0.0014** | 0.0024** | 0.1227 |
| **CXCL1** |  |  |  |  |  |  |  |
| *Mann-Whitney Utest* |  |  |  |  |  |  |  |
| U test | 12 | 6 | 4 | 21.5 | 1 | 0 | 2 |
| P-value | 1 | **0.021*** | **0.0341*** | 0.749 | **0.048*** | **0.010**** | **0.0101*** |
| *Unpaired t test* |  |  |  |  |  |  |  |
| P value | 0.8616 | **0.013*** | **0.0136*** | 0.9557 | **0.0476*** | **0.0090**** | **0.0078**** |
| t, df | t=0.1801 df=8 | t=2.913 df=12 | t=2.988 df=10 | t=0.05677 df=12 | t=2.483 df=6 | t=4.462 df=6 | t=3.315 df=10 |
| Difference between means | 1.960 ± 10.89 | -20.44 ± 7.017 | -29.07 ± 9.729 | 0.5971 ± 10.52 | -36.19 ± 14.57 | -186.0 ± 75.54 | -159.0 ± 47.98 |
| 95% confidence interval | -23.14 to 27.06 | -35.73 to -5.153 | -50.75 to -7.396 | -22.32 to 23.52 | -71.85 to -0.5311 | -370.8 to -1.164 | -265.9 to -52.14 |
| R squared | 0.004036 | 0.4143 | 0.4717 | 0.0002685 | 0.5069 | 0.5026 | 0.5235 |
| *F test to compare variances* |  |  |  |  |  |  |  |
| F,DFn, Dfd | 2.095, 4, 4 | 1.475, 6, 6 | 3.456, 4, 6 | 2.771, 6, 6 | 2.073, 4, 2 | 68.64, 4, 2 | 27.99, 4, 6 |
| P value | 0.4915 | 0.6489 | 0.1715 | 0.2404 | 0.7017 | 0.0288* | 0.0010** |
| **CXCL12** |  |  |  |  |  |  |  |
| *Mann-Whitney Utest* |  |  |  |  |  |  |  |
| U test | 11.5 | 0 | 0 | 1 | 0 | 0 | 1 |
| P-value | 0.9161 | **0.0159*** | **0.0079**** | **0.0256*** | **0.0010**** | **0.0015**** | **0.0317*** |
| *Unpaired t test* |  |  |  |  |  |  |  |
| P value | 0.7342 | **0.0088**** | **0.0005***** | **0.0335*** | **0.0031**** | **0.0003***** | **0.0087**** |
| t, df | t=0.3516 df=8 | t=3.598 df=7 | t=5.629 df=8 | t=2.429 df=11 | t=3.696 df=12 | t=6.597 df=7 | t=3.600 df=7 |
| Difference between means | -2.122 ± 6.036 | -28.75 ± 7.990 | -64.10 ± 11.39 | -76.94 ± 31.67 | -103.6 ± 28.02 | -155.6 ± 23.58 | -93.57 ± 26.00 |
| 95% confidence interval | -16.04 to 11.80 | -47.64 to -9.852 | -90.36 to -37.84 | -146.6 to -7.229 | -164.6 to -42.50 | -211.3 to -99.79 | -155.1 to -32.09 |
| R squared | 0.01522 | 0.6491 | 0.7984 | 0.3492 | 0.5323 | 0.8615 | 0.6492 |
| *F test to compare variances* |  |  |  |  |  |  |  |
| F,DFn, Dfd | 1.266, 4, 4 | 1.920, 3, 4 | 5.372, 4, 4 | 19.18, 8, 3 | 46.60, 8, 4 | 34.52, 3, 4 | 5.078, 3, 4 |
| P value | 0.8250 | 0.5359 | 0.1323 | 0.0337* | 0.0022** | 0.0051** | 0.1505 |
| **CXCL9** |  |  |  |  |  |  |  |
| *Mann-Whitney Utest* |  |  |  |  |  |  |  |
| U test | 10.50 | 2 | 0 | 1 | 0 | 0 | 14 |
| P-value | 0.753 | **0.0317*** | **0.0016**** | **0.0131*** | **0.0016**** | **0.0159*** | 0.8081 |
| *Unpaired t test* |  |  |  |  |  |  |  |
| P value | 0.7183 | **0.0201*** | **0.0015**** | **0.0181*** | **0.0011**** | **0.0051**** | 0.9566 |
| t, df | t=0.3738 df=8 | t=2.892 df=8 | t=4.185 df=11 | t=2.228 df=11 | t=4.353 df=11 | t=4.009 df=7 | t=0.05579 df=10 |
| Difference between means | 128.9 ± 344.9 | -821.0 ± 283.8 | -3367 ± 804.6 | -2546 ± 788.9 | -3496 ± 803.2 | -3557 ± 887.3 | -60.91 ± 1092 |
| 95% confidence interval | -666.5 to 924.3 | -1475 to -166.5 | -5138 to -1596 | -4283 to -809.9 | -5264 to -1728 | -5656 to -1459 | -2493 to 2371 |
| R squared | 0.01717 | 0.5112 | 0.6142 | 0.4864 | 0.6327 | 0.6966 | 0.0003112 |
| *F test to compare variances* |  |  |  |  |  |  |  |
| F,DFn, Dfd | 1.069, 4, 4 | 3.222, 4, 4 | 9.613, 7, 4 | 30.97, 7, 4 | 10.28, 7, 4 | 12.87, 3, 4 | 1.252, 3, 7 |
| P value | 0.9499 | 0.2835 | 0.0451* | 0.0050** | 0.0400* | 0.0320+ | 0.7231 |
| **HGF** |  |  |  |  |  |  |  |
| *Mann-Whitney Utest* |  |  |  |  |  |  |  |
| U test | 3 | 13 | 0 | 0 | 0 | 0 | 5 |
| P-value | **0.049*** | 0.9273 | **0.0020**** | **0.0020**** | **0.0099**** | **0.0025**** | **0.0127*** |
| *Unpaired t test* |  |  |  |  |  |  |  |
| P value | **0.0432*** | 0.8996 | **0.0006***** | **0.0001***** | **0.0051**** | **P<0.0001** | **0.0036**** |
| t, df | t=1.942 df=7 | t=0.1298 df=9 | t=4.638 df=12 | t=4.716 df=20 | t=2.151 df=20 | t=6.248 df=10 | t=3.545 df=13 |
| Difference between means | -294.7 ± 151.7 | 10.26 ± 79.02 | -1285 ± 277.0 | -1760 ± 373.1 | -1576 ± 379.7 | -2506 ± 401.0 | -1394 ± 393.3 |
| 95% confidence interval | -653.5 to 64.11 | -168.5 to 189.0 | -1888 to -681.2 | -2538 to -981.4 | -2368 to -784.1 | -3399 to -1612 | -2243 to -544.6 |
| R squared | 0.3502 | 0.001869 | 0.6419 | 0.5266 | 0.4628 | 0.7961 | 0.4915 |
| *F test to compare variances* |  |  |  |  |  |  |  |
| F,DFn, Dfd | 7.153, 4, 3 | 1.605, 6, 3 | 25.47, 9, 3 | 51.78, 14, 6 | 9.644, 14, 6 | 10.51, 4, 6 | 3.557, 4, 9 |
| P value | 0.1378 | 0.7487 | 0.0222* | P<0.0001*** | 0.0107* | 0.0141* | 0.1054 |
| **IFN-α** |  |  |  |  |  |  |  |
| *Mann-Whitney Utest* |  |  |  |  |  |  |  |
| U test | 6 | 4 | 0 | 0 | 0 | 0 | 0 |
| P-value | 0.6857 | 0.1905 | **0.011*** | **0.012*** | **0.0014**** | **0.0059**** | **0.0158*** |
| *Unpaired t test* |  |  |  |  |  |  |  |
| P value | 0.5591 | 0.1610 | **0.0092**** | **0.0063**** | **0.0060**** | **0.0001***** | **0.0011**** |
| t, df | t=0.6184 df=6 | t=1.568 df=7 | t=7.143 df=12 | t=3.116 df=17 | t=3.136 df=17 | t=7.595 df=7 | t=6.856 df=12 |
| Difference between means | -0.5725 ± 0.9258 | -1.781 ± 1.136 | -53.09 ± 7.433 | -83.18 ± 26.69 | -83.70 ± 26.69 | -163.4 ± 21.51 | -111.5 ± 16.27 |
| 95% confidence interval | -2.838 to 1.693 | -4.468 to 0.9061 | -69.29 to -36.90 | -139.5 to -26.86 | -140.0 to -27.38 | -214.2 to -112.5 | -147.0 to -76.08 |
| R squared | 0.05991 | 0.2598 | 0.8096 | 0.3636 | 0.3665 | 0.8918 | 0.7966 |
| *F test to compare variances* |  |  |  |  |  |  |  |
| F,DFn, Dfd | 1.798, 3, 3 | 1.528, 4, 3 | 95.20, 9, 3 | 1019, 13, 4 | 1048, 13, 4 | 731.0, 3, 4 | 11.41, 3, 9 |
| P value | 0.6417 | 0.7578 | 0.0032** | P<0.0001*** | P<0.0001*** | P<0.0001*** | 0.0040** |
| **IL-16** |  |  |  |  |  |  |  |
| *Mann-Whitney Utest* |  |  |  |  |  |  |  |
| U test | 12 | 1 | 0 | 0 | 0 | 0 | 3 |
| P-value | 1 | **0.0159*** | **0.0079**** | **0.012*** | **0.0022**** | **0.0043**** | **0.0303*** |
| *Unpaired t test* |  |  |  |  |  |  |  |
| P value | 0.8743 | **0.0139*** | **P<0.0001***** | **0.0181*** | **0.0022**** | **0.0008***** | **0.0225*** |
| t, df | t=0.1633 df=8 | t=3.137 df=8 | t=8.187 df=8 | t=2.085 df=14 | t=3.746 df=14 | t=4.965 df=9 | t=2.750 df=9 |
| Difference between means | 5.062 ± 31.00 | -109.0 ± 34.74 | -378.0 ± 46.17 | -537.4 ± 174.2 | -651.5 ± 173.9 | -875.1 ± 176.2 | -492.1 ± 178.9 |
| 95% confidence interval | -66.42 to 76.54 | -189.1 to -28.87 | -484.5 to -271.5 | -911.1 to -163.7 | -1025 to -278.4 | -1274 to -476.5 | -896.8 to -87.32 |
| R squared | 0.003323 | 0.5516 | 0.8934 | 0.4047 | 0.5006 | 0.7326 | 0.4566 |
| *F test to compare variances* |  |  |  |  |  |  |  |
| F,DFn, Dfd | 1.041, 4, 4 | 1.462, 4, 4 | 3.350, 4, 4 | 40.36, 10, 4 | 61.45, 10, 4 | 63.99, 5, 4 | 18.35, 5, 4 |
| P value | 0.9697 | 0.7217 | 0.2685 | 0.0028** | 0.0012** | 0.0013** | 0.0146* |
| **IL-18** |  |  |  |  |  |  |  |
| *Mann-Whitney Utest* |  |  |  |  |  |  |  |
| U test | 1 | 0 | 0 | 0 | 0 | 0 | 3 |
| P-value | **0.0317*** | **0.0159*** | **0.0095**** | **0.011*** | **0.012*** | **0.0001***** | **0.0152*** |
| *Unpaired t test* |  |  |  |  |  |  |  |
| P value | **0.0191*** | **0.0127*** | **0.0002***** | **0.0041**** | **0.0073**** | **0.0001***** | **0.0056**** |
| t, df | t=3.029 df=7 | t=3.323 df=7 | t=6.648 df=8 | t=3.455 df=9 | t=3.035 df=9 | t=6.391 df=9 | t=3.512 df=10 |
| Difference between means | -63.12 ± 20.84 | -55.96 ± 16.84 | -239.7 ± 36.05 | -183.7 ± 33.68 | -176.6 ± 35.07 | -387.8 ± 60.69 | -211.3 ± 60.16 |
| 95% confidence interval | -112.4 to -13.84 | -95.80 to -16.13 | -322.8 to -156.5 | -259.9 to -107.5 | -255.9 to -97.23 | -525.1 to -250.6 | -345.3 to -77.23 |
| R squared | 0.5673 | 0.6120 | 0.8467 | 0.7678 | 0.7380 | 0.8194 | 0.5522 |
| *F test to compare variances* |  |  |  |  |  |  |  |
| F,DFn, Dfd | 5.899, 4, 3 | 3.595, 4, 3 | 19.05, 5, 3 | 5.300, 5, 4 | 3.230, 5, 4 | 11.27, 5, 4 | 3.489, 5, 5 |
| P value | 0.1766 | 0.3214 | 0.0352* | 0.1313 | 0.2790 | 0.0359* | 0.1964 |
| **IL-2R** |  |  |  |  |  |  |  |
| *Mann-Whitney Utest* |  |  |  |  |  |  |  |
| U test | 2 | 0 | 0 | 0 | 0 | 0 | 0 |
| P-value | **0.0317*** | **0.017*** | **0.0016**** | **0.011*** | **0.012*** | **0.0043**** | **0.011*** |
| *Unpaired t test* |  |  |  |  |  |  |  |
| P value | **0.0437*** | **0.0121*** | **0.0001***** | **0.0026**** | **0.0013**** | **0.0007***** | **0.0022**** |
| t, df | t=2.392 df=8 | t=3.226 df=8 | t=5.902 df=11 | t=3.878 df=11 | t=3.281 df=11 | t=5.005 df=9 | t=3.344 df=12 |
| Difference between means | -70.82 ± 29.60 | -94.65 ± 29.34 | -320.6 ± 54.33 | -226.0 ± 58.28 | -249.8 ± 58.36 | -1552 ± 310.0 | -1302 ± 243.6 |
| 95% confidence interval | -139.1 to -2.557 | -162.3 to -27.00 | -440.2 to -201.1 | -354.3 to -97.72 | -378.3 to -121.4 | -2253 to -850.4 | -1833 to -771.0 |
| R squared | 0.4171 | 0.5654 | 0.7600 | 0.5775 | 0.6249 | 0.7357 | 0.7041 |
| *F test to compare variances* |  |  |  |  |  |  |  |
| F,DFn, Dfd | 15.21, 4, 4 | 14.92, 4, 4 | 52.22, 7, 4 | 3.501, 7, 4 | 3.434, 7, 4 | 114.0, 5, 4 | 33.19, 5, 7 |
| P value | 0.0219* | 0.0227* | 0.0018** | 0.2432 | 0.2503 | 0.0004*** | 0.0002*** |
| **Leptin** |  |  |  |  |  |  |  |
| *Mann-Whitney Utest* |  |  |  |  |  |  |  |
| U test | 0 | 0 | 0 | 2 | 0 | 0 | 0 |
| P-value | **0.0043**** | **0.0022**** | **0.0043**** | **0.0435*** | **0.0079**** | **0.00357**** | **0.0357*** |
| *Unpaired t test* |  |  |  |  |  |  |  |
| P value | **P<0.0001***** | **0.0002***** | **0.0002***** | **0.0413*** | **0.0003***** | **0.0001***** | **0.0020**** |
| t, df | t=11.88 df=9 | t=5.731 df=10 | t=6.062 df=9 | t=2.494 df=7 | t=6.114 df=8 | t=8.810 df=6 | t=5.204 df=6 |
| Difference between means | -44780 ± 3770 | -32660 ± 5698 | -15450 ± 2548 | 9977 ± 4000 | 29330 ± 4798 | 47760 ± 5421 | 18430 ± 3541 |
| 95% confidence interval | -53310 to -36250 | -45350 to -19960 | -21210 to -9683 | 517.3 to 19440 | 18270 to 40400 | 34500 to 61030 | 9765 to 27100 |
| R squared | 0.9400 | 0.7666 | 0.8033 | 0.4706 | 0.8237 | 0.9282 | 0.8186 |
| *F test to compare variances* |  |  |  |  |  |  |  |
| F,DFn, Dfd | 16.98, 4, 5 | 39.71, 5, 5 | 7.075, 4, 5 | 1.117, 3, 4 | 2.399, 4, 4 | 28.66, 4, 2 | 11.94, 4, 2 |
| P value | 0.0082** | 0.0010** | 0.0546 | 0.8820 | 0.4174 | 0.0680 | 0.1575 |
| **PECAM-1** |  |  |  |  |  |  |  |
| *Mann-Whitney Utest* |  |  |  |  |  |  |  |
| U test | 19 | 1 | 1 | 6 | 2 | 2 | 5 |
| P-value | 0.8857 | **0.0471*** | **0.0471*** | 0.6857 | **0.0423*** | **0.0452*** | **0.4857** |
| *Unpaired t test* |  |  |  |  |  |  |  |
| P value | 0.9520 | **0.0454*** | **0.0254*** | 0.6603 | **0.04905*** | **0.0458*** | 0.6362 |
| t, df | t=0.06274 df=6 | t=2.518 df=6 | t=2.955 df=6 | t=0.4621 df=6 | t=1.916 df=9 | t=1.800 df=9 | t=0.4981 df=6 |
| Difference between means | 385.4 ± 6142 | -13450 ± 5341 | -15790 ± 5343 | -2343 ± 5070 | -9139 ± 6456 | -11760 ± 6533 | -2618 ± 5257 |
| 95% confidence interval | -14650 to 15420 | -26520 to -376.7 | -28860 to -2716 | -14750 to 10060 | -23740 to 5464 | -26540 to 3020 | -15480 to 10250 |
| R squared | 0.0006556 | 0.5137 | 0.5928 | 0.03436 | 0.1821 | 0.2646 | 0.03970 |
| *F test to compare variances* |  |  |  |  |  |  |  |
| F,DFn, Dfd | 1.406, 3, 3 | 1.221, 3, 3 | 1.219, 3, 3 | 1.001, 3, 3 | 2.594, 6, 3 | 2.258, 6, 3 | 1.149, 3, 3 |
| P value | 0.7863 | 0.8735 | 0.8743 | 0.9992 | 0.4647 | 0.5381 | 0.9119 |
| **Prolactin** |  |  |  |  |  |  |  |
| *Mann-Whitney Utest* |  |  |  |  |  |  |  |
| U test | 39 | 39 | 2 | 0 | 0 | 0 | 8 |
| P-value | 0.3653 | 0.3653 | **0.012*** | **0.0159*** | **0.0159*** | **0.0317*** | 0.7302 |
| *Unpaired t test* |  |  |  |  |  |  |  |
| P value | 0.3100 | 0.3042 | **0.0059**** | **0.0076**** | **0.0102*** | **0.0111*** | 0.8240 |
| t, df | t=1.043 df=19 | t=1.056 df=19 | t=3.406 df=11 | t=3.703 df=7 | t=2.484 df=7 | t=2.420 df=7 | t=0.2309 df=7 |
| Difference between means | -4721 ± 4526 | -4744 ± 4492 | 17920 ± 5262 | 13830 ± 3735 | 13780 ± 3956 | 15330 ± 4482 | 817.8 ± 3542 |
| 95% confidence interval | -14200 to 4752 | -14150 to 4658 | 6338 to 29500 | 4996 to 22660 | 4427 to 23140 | 4729 to 25930 | -7559 to 9195 |
| R squared | 0.05416 | 0.05545 | 0.5132 | 0.6620 | 0.6343 | 0.6256 | 0.007558 |
| *F test to compare variances* |  |  |  |  |  |  |  |
| F,DFn, Dfd | 1.013, 7, 12 | 1.029, 12, 7 | 5.107, 8, 3 | 2.900, 3, 4 | 3.416, 3, 4 | 1.698, 3, 4 | 1.787, 4, 3 |
| P value | 0.9374 | 1.0151 | 0.2073 | 0.3303 | 0.2662 | 0.6084 | 0.6610 |
| **sIL-6Ra** |  |  |  |  |  |  |  |
| *Mann-Whitney Utest* |  |  |  |  |  |  |  |
| U test | 2 | 0 | 0 | 0 | 2 | 0 | 1 |
| P-value | **0.0456*** | **0.0015**** | **0.0159*** | **0.035*** | **0.0317*** | **0.0001***** | **0.0317*** |
| *Unpaired t test* |  |  |  |  |  |  |  |
| P value | **0.0241*** | **0.0060**** | **0.0017**** | **0.0304*** | **0.0060**** | **0.0002***** | **0.0084**** |
| t, df | t=2.866 df=7 | t=3.890 df=7 | t=4.945 df=7 | t=2.705 df=7 | t=3.704 df=8 | t=7.350 df=7 | t=3.634 df=7 |
| Difference between means | -18080 ± 6309 | -23900 ± 6146 | -57580 ± 11640 | -32130 ± 11880 | -39490 ± 10660 | -100700 ± 13700 | -61170 ± 16830 |
| 95% confidence interval | -33000 to -3163 | -38440 to -9370 | -85120 to -30040 | -60220 to -4042 | -64080 to -14910 | -133100 to -68270 | -101000 to -21360 |
| R squared | 0.5400 | 0.6837 | 0.7774 | 0.5111 | 0.6317 | 0.8853 | 0.6535 |
| *F test to compare variances* |  |  |  |  |  |  |  |
| F,DFn, Dfd | 1.292, 4, 3 | 1.188, 4, 3 | 6.206, 4, 3 | 4.514, 4, 3 | 4.804, 4, 4 | 8.597, 3, 4 | 1.790, 3, 4 |
| P value | 0.8678 | 0.9244 | 0.1656 | 0.2459 | 0.1577 | 0.0645 | 0.5768 |
| **VEGFR-1** |  |  |  |  |  |  |  |
| *Mann-Whitney Utest* |  |  |  |  |  |  |  |
| U test | 0 | 0 | 0 | 7 | 0 | 0 | 4 |
| P-value | **0.00154**** | **0.0286*** | **0.0286*** | 0.8857 | **0.0011**** | **0.0011**** | 0.342 |
| *Unpaired t test* |  |  |  |  |  |  |  |
| P value | **P<0.0001***** | **0.0028**** | **0.0056**** | 0.9026 | **0.0022**** | **0.0086**** | 0.3256 |
| t, df | t=7.977 df=7 | t=4.877 df=6 | t=4.206 df=6 | t=0.1276 df=6 | t=4.713 df=7 | t=3.613 df=7 | t=1.070 df=6 |
| Difference between means | -1338 ± 167.8 | -467.2 ± 95.79 | -483.1 ± 114.8 | -15.82 ± 124.0 | 855.2 ± 181.5 | 689.6 ± 190.9 | -165.6 ± 154.7 |
| 95% confidence interval | -1735 to -941.5 | -701.6 to -232.8 | -764.1 to -202.0 | -319.3 to 287.6 | 426.0 to 1284 | 238.2 to 1141 | -544.1 to 212.9 |
| R squared | 0.9009 | 0.7986 | 0.7468 | 0.002707 | 0.7604 | 0.6509 | 0.1604 |
| *F test to compare variances* |  |  |  |  |  |  |  |
| F,DFn, Dfd | 7.083, 4, 3 | 1.627, 3, 3 | 2.776, 3, 3 | 1.706, 3, 3 | 2.552, 4, 3 | 1.738, 4, 3 | 1.468, 3, 3 |
| P value | 0.1396 | 0.6990 | 0.4241 | 0.6716 | 0.4675 | 0.6778 | 0.7599 |
| **VEGFR-2** |  |  |  |  |  |  |  |
| *Mann-Whitney Utest* |  |  |  |  |  |  |  |
| U test | 0 | 0 | 0 | 7 | 0 | 0 | 0 |
| P-value | **0.0001***** | **0.0286*** | **0.0159*** | 0.555 | **0.0099**** | **0.0286*** | 0.998 |
| *Unpaired t test* |  |  |  |  |  |  |  |
| P value | **0.0006***** | **0.0024**** | **0.0004***** | 0.6043 | **0.0048**** | **0.0085**** | 0.6674 |
| t, df | t=6.652 df=6 | t=5.032 df=6 | t=6.379 df=7 | t=0.5426 df=7 | t=4.064 df=7 | t=3.850 df=6 | t=0.4484 df=7 |
| Difference between means | -14180 ± 2132 | -5429 ± 1079 | -6066 ± 950.8 | -636.7 ± 1174 | 8113 ± 1996 | 8617 ± 2238 | 504.1 ± 1124 |
| 95% confidence interval | -19390 to -8963 | -8069 to -2789 | -8314 to -3817 | -3412 to 2139 | 3392 to 12830 | 3140 to 14090 | -2155 to 3163 |
| R squared | 0.8806 | 0.8085 | 0.8532 | 0.04035 | 0.7024 | 0.7118 | 0.02792 |
| *F test to compare variances* |  |  |  |  |  |  |  |
| F,DFn, Dfd | 15.51, 3, 3 | 3.229, 3, 3 | 2.444, 4, 3 | 1.321, 3, 4 | 6.346, 3, 4 | 5.757, 3, 3 | 1.102, 3, 4 |
| P value | 0.0497 | 0.3615 | 0.4888 | 0.7687 | 0.1063 | 0.1845 | 0.8910 |
